# The NOXA–MCL1–BIM axis defines lifespan on extended mitotic arrest

**DOI:** 10.1038/ncomms7891

**Published:** 2015-04-29

**Authors:** Manuel D. Haschka, Claudia Soratroi, Susanne Kirschnek, Georg Häcker, Richard Hilbe, Stephan Geley, Andreas Villunger, Luca L. Fava

**Affiliations:** 1 Division of Developmental Immunology, Biocenter, Medical University Innsbruck, Innrain 80-82, A-6020 Innsbruck, Austria; 2 Institute for Medical Microbiology and Hygiene, University Medical Center Freiburg, 79106 Freiburg, Germany; 3 Division of Molecular Pathophysiology, Biocenter, Medical University Innsbruck, Innrain 80-82, A-6020 Innsbruck, Austria

## Abstract

Cell death on extended mitotic arrest is considered arguably most critical for the efficacy of microtubule-targeting agents (MTAs) in anticancer therapy. While the molecular machinery controlling mitotic arrest on MTA treatment, the spindle assembly checkpoint (SAC), appears well defined, the molecular components executing cell death, as well as factors connecting both networks remain poorly understood. Here we conduct a mini screen exploring systematically the contribution of individual BCL2 family proteins at single cell resolution to death on extended mitotic arrest, and demonstrate that the mitotic phosphorylation of BCL2 and BCLX represent a priming event for apoptosis that is ultimately triggered by NOXA-dependent MCL1 degradation, enabling BIM-dependent cell death. Our findings provide a comprehensive model for the initiation of apoptosis in cells stalled in mitosis and provide a molecular basis for the increased efficacy of combinatorial treatment of cancer cells using MTAs and BH3 mimetics.

## Abstract (web summary)

Cells experiencing extended mitotic arrest often undergo cell death as a result of steadily declining levels of the apoptotic inhibitor MCL1, but the mechanism controlling this process is poorly understood. Here, Haschka *et al.* show that the BH3-only protein NOXA promotes the degradation of MCL1, enabling BIM-dependent cell death.

## Graphical abstract

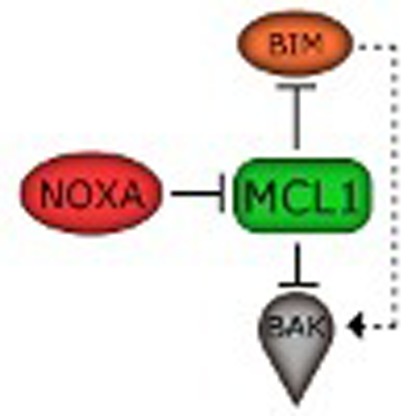

Microtubule-targeting agents (MTAs) such as taxol and vinca alkaloids prove clinically effective in eradicating tumours and are standard of care for decades, for example, in the treatment of breast or lung cancer^1^. MTAs, however, severely interfere not only with the mitotic functions of microtubules, resulting in a cell division blockade, but also with their critical non-mitotic functions, for example, in vesicle transport. The major limitations of these agents in clinical use are neurotoxicity that patients experience on prolonged treatment, reflecting cytotoxicity towards non-dividing cells and tissues with low cellular turnover^2^,^3^, sparking the search for novel cell division inhibitors^4^.

While new compounds targeting, for example, the mitotic kinesin Eg5 or the kinase Plk1, have been developed, their clinical efficacy appears limited^5^,^6^, fuelling a debate on whether MTAs achieve clinical benefit by directly killing dividing cancer cells or rather act on non-dividing cells^7^,^8^,^9^,^10^,^11^.Whereas it is unlikely that this controversy will be solved quickly, due to inherent paradoxes^12^, the fact that mitotic arrest can trigger cell death remains undisputed^13^. Understanding the molecular mechanism of death in mitosis will certainly help to improve MTA efficacy, for example, in combinatorial treatment regimens, and to better define and manage undesired clinical side effects^14^.

Cancer cells treated with anti-mitotic drugs in culture respond by displaying a highly variable phenotype that encompasses the extension of the normal cell division process^13^, mediated by the activation of a surveillance mechanism referred to as the spindle assembly checkpoint (SAC), reviewed in ref. 15. The SAC inhibits the anaphase promoting complex or cyclosome (APC/C), reviewed in ref. 16, the E3 ubiquitin ligase to Cyclin B that controls the time to mitotic exit. Mitotically arrested cells display one of two mutually exclusive fates, a phenomenon known as checkpoint adaptation or mitotic slippage, in which cells are unable to maintain the mitotic status but return to interphase without cellular division or, alternatively, cell death during mitosis. Recent work has shown that the two fates are in direct competition with each other and mitotic slippage can be seen as a putative death-escape and drug-resistance mechanisms, whereas mitotic cell death does prevent slippage, acting as a barrier against aneuploidy^17^,^18^,^19^,^20^.

In contrast to slippage, cell death in mitosis is poorly understood. Extended mitotic arrest (M-arrest) has been shown to deeply impact on the apoptotic machinery as virtually all BCL2 family proteins and initiating caspases undergo post-translational modifications, most frequently CDK1/Cyclin B-mediated phosphorylation, resulting in modulation of their activity during mitosis, reviewed in ref. 21. Among all modifications described, one stands out for the ability to relay a death signal of increasing strength on extended mitotic duration, that is, the steady decline of the apoptotic inhibitor MCL1 (refs 22, 23).

While MCL1 degradation rapidly qualified as a key component of the ‘timer' defining the mitotic lifespan of MTA-treated tumour cells, the molecular components of this mechanism remain to be elucidated in full. On the one hand, the machinery leading to MCL1 degradation remains debated and is possibly highly redundant, including different E3 ligases, such as the MULE, the APC/C and/or SCF/FBW7 (refs 22, 23, 24), while on the other hand the contribution of other BCL2 pro-survival family proteins on neutralization of MCL1, particularly of the BH3-only subfamily, remains poorly understood. Recent evidence proposes priming roles for the BH3-only protein BID^25^, a BCL2 family effector linking extrinsic with intrinsic cell death routes on death receptor activation, and BIM, a target of mitotic kinases and direct APC^Cdc20^ and/or SCF-βTrcp substrate, implicated repeatedly, but not undisputedly, in MTA-mediated tumour cell killing^24^,^26^,^27^.

Here we aimed to systematically assess the relative contribution of individual BCL2 family members to cell death initiation on MTA treatment with single cell resolution. Our work shows that BCL2 and BCLX phosphorylation represents a priming event in mitotic cell death that is triggered by NOXA-dependent MCL1 degradation. The MCL1 decay allows in turn BIM-dependent cell death.

## Results

### Mitochondrial apoptosis mediates cell death during M-arrest

**Figure 1:**
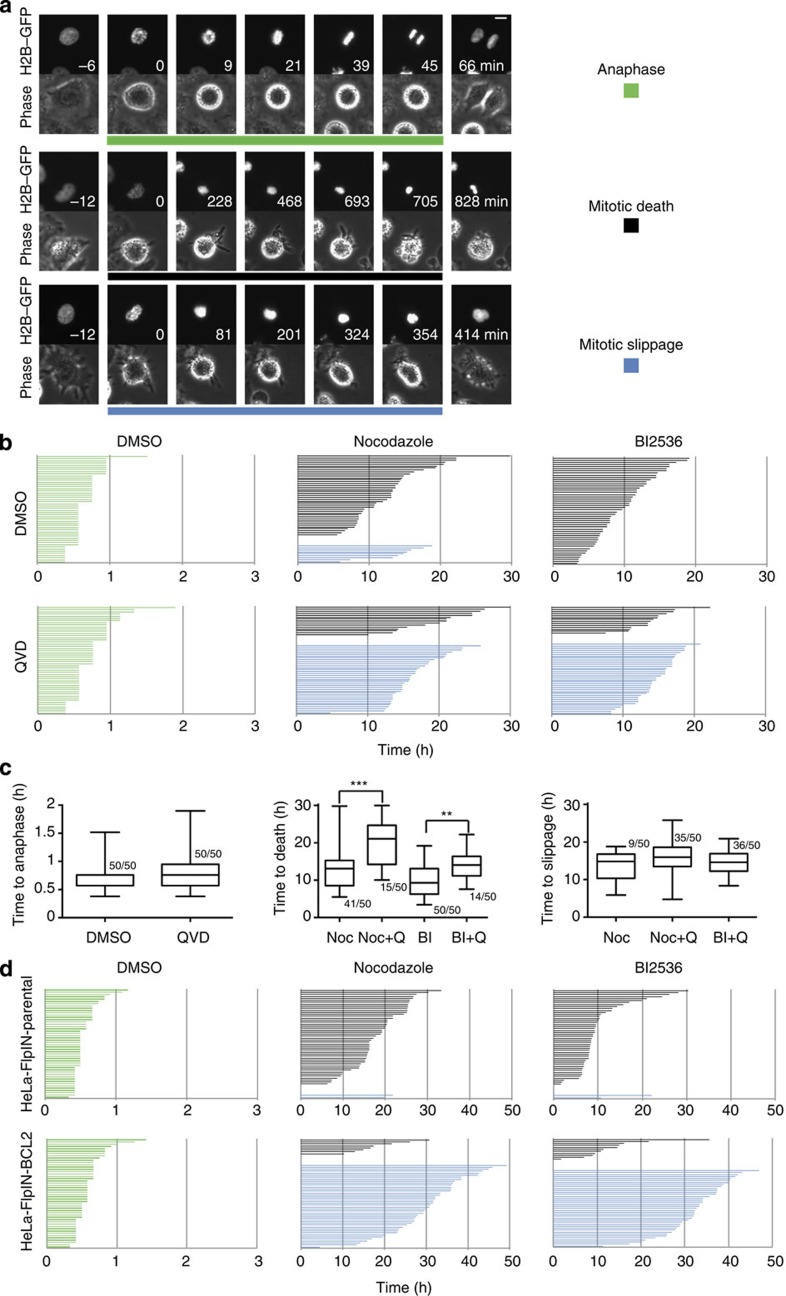
Activation of mitochondrial apoptosis on chronic SAC activation in HeLa cells. (**a**) Movie stills from HeLaS3-H2B–GFP cells subjected to time-lapse video microscopy following exposure to DMSO (upper panel), Nocodazole (central panel) and Nocodazole in combination with reversine (lower panel). The coloured line defines beginning of mitosis at nuclear envelope breakdown (NEBD) and end of the line matches the end of mitosis reflecting anaphase (green), death (black) and slippage (blue). Time in minutes is indicated. Scale bar, 10 μM. (**b**) Fate profiles of 50 HeLaS3-H2B–GFP cells exposed to DMSO, Nocodazole or BI2536 in the absence (DMSO) or presence of the pan-caspase inhibitor QVD. Colour-coding according to **a**. Time in hours is indicated. (**c**) Box (interquartile range) and whisker (min to max) plots showing the elapsed time (h) between NEBD and the indicated fate for individual cells after treatment. The fraction (X/50 events) of cells undergoing the fate of interest is indicated. Noc, Nocodazole; Q, QVD; BI, BI2536. **P<0.01; ***P<0.001 using Mann–Whitney test. (**d**) Fate profiles of HeLa-FlpIN cells transgenic for BCL2-WT subjected to treatment with DMSO, Nocodazole or BI2536. Time in hours is indicated. Both parental (upper panel) and BCL2 transgenic (lower panel) cell lines were pre-treated with 200 ng ml^−1^ doxycycline for 24 h.

Mitotic cell death or mitotic slippage followed by death cannot be distinguished when employing endpoint-based population assays usually used in cell death research. Therefore, we utilized live cell imaging as a method to score (1) fate distribution and (2) time elapsed in mitosis for individual cells undergoing extended mitotic arrest. To this end, we employed HeLa cells overexpressing fluorescently labelled histone H2B that allowed us to follow chromatin behaviour from mitosis onset, defined as the time of nuclear envelope breakdown becoming evident, till either anaphase, mitotic death or mitotic slippage (Fig. 1a). HeLa cells were chosen as a suitable system as mitotic death is the most prominent fate displayed on extended mitotic (M) arrest in these cells^17^. As triggers for M-arrest, we used Nocodazole, preventing mitotic spindle formation, leading to maximal SAC activation^28^ or the Plk1 inhibitor BI2536 that leads to SAC activation by different means, that is, by generating defects in kinetochore-to-microtubule attachments^29^.

Nocodazole treatment led to an extension of the mitotic duration >1 order of magnitude (Fig. 1b,c) and the majority of the cells underwent cell death during mitosis while only a fraction underwent mitotic slippage (Fig. 1b). Similarly, Plk1 inhibition with BI2536 triggered cell death with even higher penetrance and faster kinetics (Fig. 1b,c), highlighting the fact that inhibition of Plk1 is a better inducer of mitotic death than microtubule depolymerization, at least in HeLa cells. The addition of the pan-caspase inhibitor Q-VD-OPh (QVD) left normal mitosis largely unaffected but triggered a major switch in the distribution of cell fates on extended mitotic arrest by increasing the proportion of cells undergoing mitotic slippage on Nocodazole or BI2536 treatment (Fig. 1b and Supplementary Movies 1–6). While the few cells that still died in mitosis took longer in doing so, the kinetics of mitotic slippage (mean time to mitotic exit) remained unaffected on pan-caspase inhibition (Fig. 1c).

Next, we defined the relative contribution of mitochondrial Caspase-9-dependent apoptosis as opposed to Caspase-8-controlled cell death. As the Caspase-8 inhibitor cFlip undergoes mitotic degradation, similarly to MCL1 (refs 30, 31), we reasoned that mitotic arrest might feed not only into the activation of the mitochondrial pathway of apoptosis, but additionally or even exclusively, into the activation of Caspase-8, for example, via Ripoptosome formation^32^,^33^. To express inhibitors of either pathway in a conditional manner, we engineered a HeLa cell line carrying a single chromosomal integration of a FRT site plus mRED-H2B as a marker for imaging^34^ and transduced it with a Tet repressor. This cell line, described hereafter as HeLa-FlpIN, allowed in turn the inducible overexpression of transgenes following rapid integration at an invariable genetic locus (Supplementary Fig. 1a,b). Overexpression of an epitope-tagged version of the viral Caspase-8 inhibitor CrmA left the process of mitotic apoptosis essentially unaffected (Supplementary Fig. 1c), but provided resistance to FAS killing (that is FasR trimerization induced by FasL, not shown). In stark contrast, overexpression of BCL2, leading to a blockade of mitochondrial apoptosis, resulted in a shift in cell fate that was comparable to the one observed on pan-caspase inhibition (Fig. 1d and Supplementary Fig. 1b).

**Figure 2:**
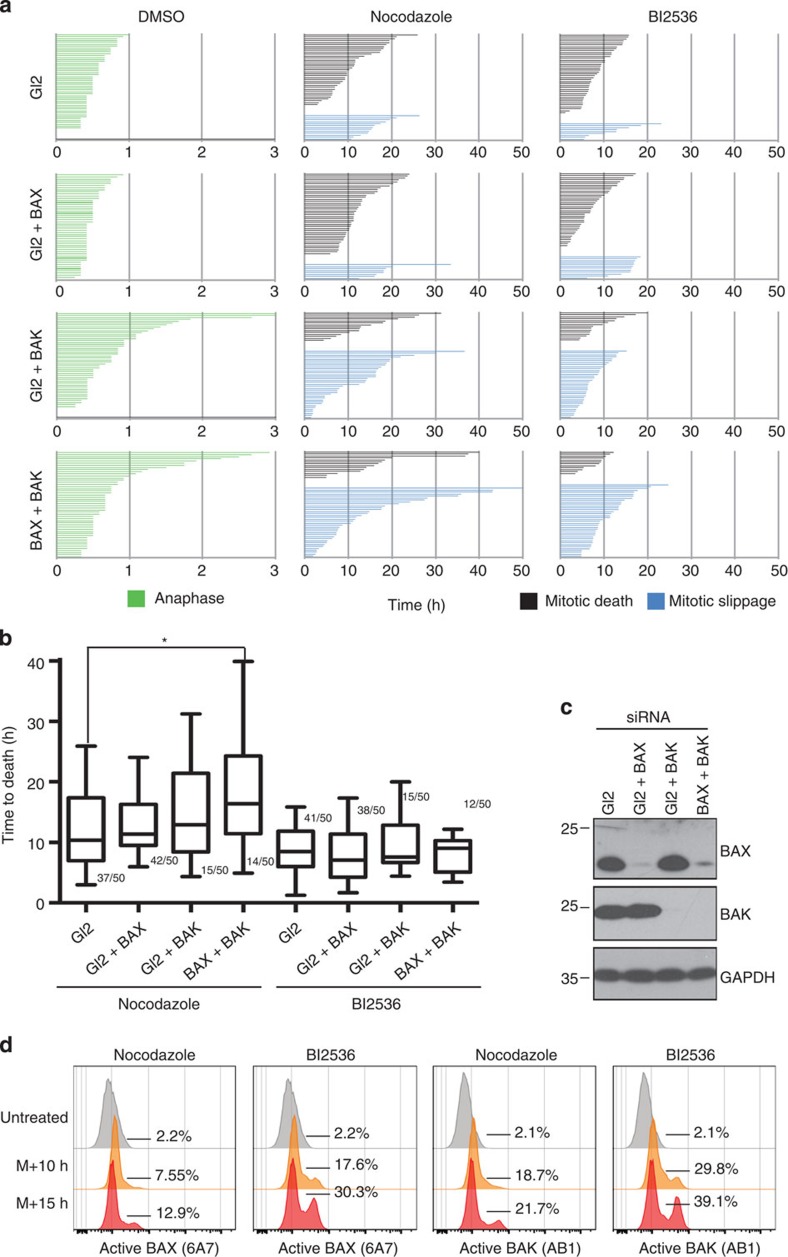
BAK is more critical than BAX for cell death induction on M-arrest in HeLa cells. (**a**) Cell fate profiles of individual cells subjected to DMSO, Nocodazole or BI2536 after transfection with the indicated siRNAs. Time in hours is indicated. (**b**) Box (interquartile range) and whisker (min to max) plots showing the elapsed time (h) between nuclear envelope breakdown (NEBD) and mitotic death for individual cells after treatments after siRNA depletion. The fraction (X/50 events) of cells undergoing the fate of interest is indicated. *P<0.05 using Mann–Whitney test. (**c**) HeLaS3-H2B–GFP cells were transfected with the constant amount of siRNA targeting BAK or BAX. A fraction of the cells were harvested and analysed by immunoblotting, while the remaining cells were re-seeded and subjected to live cell imaging **a**. (**d**) Cells were either left asynchronous and untreated or synchronized by single thymidine arrest and released into fresh medium in the presence of the indicated drugs. Cells were harvested after either 10 or 15 h of M-arrest. Cells of the indicated genotypes were stained with the indicated conformation-specific antibodies and quantified by flow cytometry. Histograms are representatives from one out of two experiments yielding similar results.

As mitochondrial apoptosis requires the activation of either BAX or BAK to trigger mitochondrial outer membrane permeabilization (MOMP)^35^,^36^, we asked whether only one of them or both are functionally important to trigger mitotic apoptosis. RNA interference (RNAi)-depletion of BAX, BAK, or both, was performed (Fig. 2c) followed by cell fate profiling. Depletion of BAX did not have a major impact on mitotic apoptosis of HeLa cells, whereas BAK depletion enhanced the fraction of HeLa cells undergoing mitotic slippage on treatment with both Nocodazole and BI2536 (Fig. 2a). The double depletion resulted in a phenotype reminiscent to the one observed on BAK depletion (Fig. 2a), but the time required to die in mitosis on Nocodazole treatment was significantly extended only on double depletion (Fig. 2b). Our data are consistent with the idea that BAK is more critical than BAX in triggering mitotic apoptosis, yet the BAK short interfering RNA (siRNA) also appeared more effective than BAX. To monitor BAX and BAK activation on extended M-arrest, we used conformation-specific antibodies and assessed intracellular staining by flow cytometry. Both BAX and BAK became gradually activated on extending the duration of M-arrest triggered by Nocodazole or BI2536 (Fig. 2d). Taken together, BAK appears to play a more prominent, yet not exclusive, role in the induction of mitotic apoptosis of HeLa cells.

### Phosphorylation of BCL2 and BCLX poorly alters functionality

**Figure 3:**
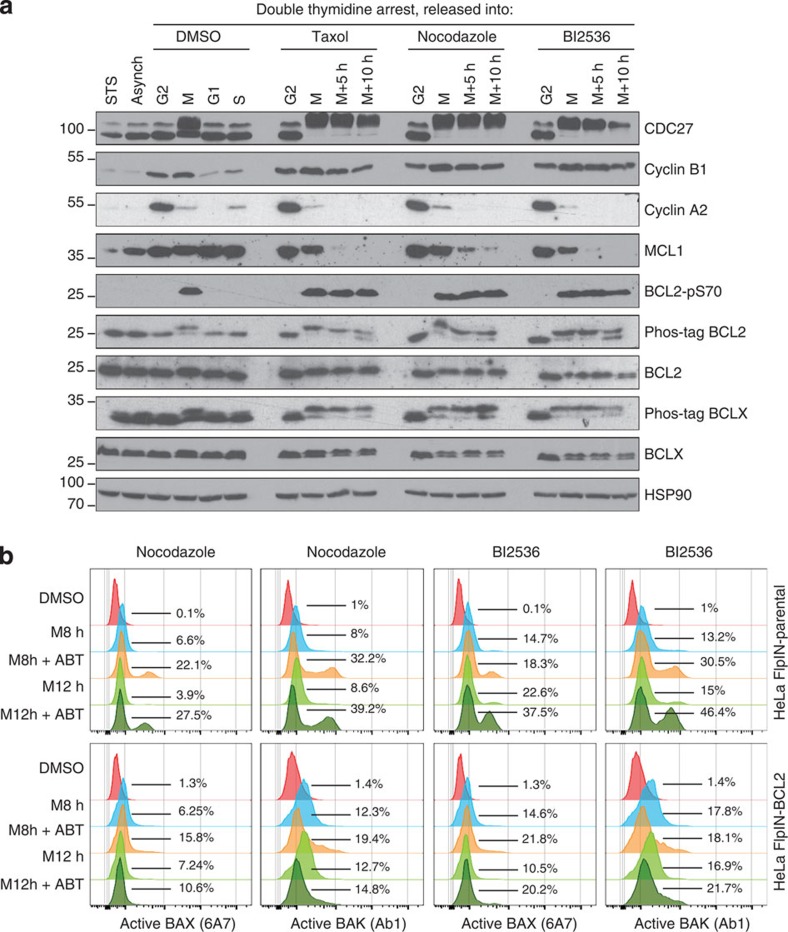
MCL1 undergoes degradation during M-arrest while BCL2 and BCLX remain active. (**a**) Cells were asynchronous and left untreated (Asynch), asynchronous and treated with staurosporine (STS) or synchronized by double-thymidine block and released from the arrest into DMSO-, Taxol-, Nocodazole- or BI2536-containing media. Cells were harvested in G2 and early M phase. Part of the mitotic cells were re-seeded in the presence of the above-mentioned compounds to be harvested 5 or 10 h later. Cells were harvested, lysed and analysed by immunoblotting. (**b**) Asynchronous cells were treated with DMSO or synchronized with single thymidine arrest, washed and released into fresh medium in the presence of the indicated drugs and harvested after either 8 or 12 h of M arrest. Cells of the indicated genotypes were stained using the indicated conformation-specific antibodies and analysed by flow cytometry. All cell lines were pre-treated with 200ng ml^−1^ doxycycline for 24h. Histograms are representatives from one out of two experiments yielding similar results.

To follow modifications of BCL2 family proteins that drive MOMP during mitotic arrest, we employed double-thymidine arrest to synchronize HeLa cells at the G1/S boundary and followed them on release in the presence of DMSO, Taxol, Nocodazole or BI2536. In all cases, mitotic cells have been harvested by selective shake-off and re-seeded in the presence of the above-mentioned drugs for variable amount of time (Fig. 3a). Analysis of the phospho-shift of CDC27, a mitotic substrate of CDK1 and immunoblot for Cyclin A2 and B1, undergoing rapid and markedly slower degradation during M-arrest, respectively, validated our synchronization protocol (Fig. 3a).

Immunoblot analysis of MCL1 revealed that protein levels steadily decreased during M-arrest, confirming previous reports^22^,^23^. In stark contrast, neither BCL2 nor BCLX were degraded during mitotic arrest but changed their electrophoretic mobility, indicative for post-translational modification (Fig. 3a). A phospho-shift affecting a large pool of both proteins became evident in phos-tag gels, detectable on early mitotic arrest and remaining constant over time (Fig. 3a). The use of phospho-specific antibodies, directed to inter-domain residues of BCL2 (Ser70) and of BCLX (Ser62) revealed that phosphorylation of both residues contributed to the above-mentioned shift (Fig. 3a and Supplementary Fig. 2a,b). Taken together, the phosphorylation of BCL2 and BCLX encompassing the inter-domain unstructured loop in both proteins is a *bona fide* mitotic event and does not vary quantitatively or qualitatively in the course of M-arrest, meaning an all or nothing event. Interestingly, when we forced mitotic slippage by the inhibition of the SAC kinase MPS1, all the above-mentioned phenomena and cleavage of PARP1 by effector caspases were overruled (Supplementary Fig. 2a,b), consistent with the notion that mitotic slippage exerts a (temporary) pro-survival function^37^.

Of note, the functional impact of BCL2 phosphorylation remains a matter of debate and has been reported to exert suppressive as well as activating influence on protein function (see for example refs 38, 39). Hence, we wondered whether inhibiting BCL2 and BCLX in the course of M-arrest by using the BH3 mimetic ABT-737, targeting BCL2 and BCLX but not MCL1, would enhance mitotic apoptosis, arguing that residual BCL2 and BCLX activity is present. To this end, we monitored BAX and BAK activation. Strikingly, M-arrest led to activation of BAX and BAK that was further enhanced by ABT-737 over time (Fig. 3b). Simultaneous overexpression of BCL2 reduced the activation of both BAX and BAK (Fig. 3b). Taken together, mitotic apoptosis in HeLa cells is triggered by MCL1 degradation that associates with the induction of BAK and BAX conformational changes, indicative for their activation, but BCL2 and BCLX retain significant activity in the course of M-arrest despite their extensive phosphorylation.

**Figure 4:**
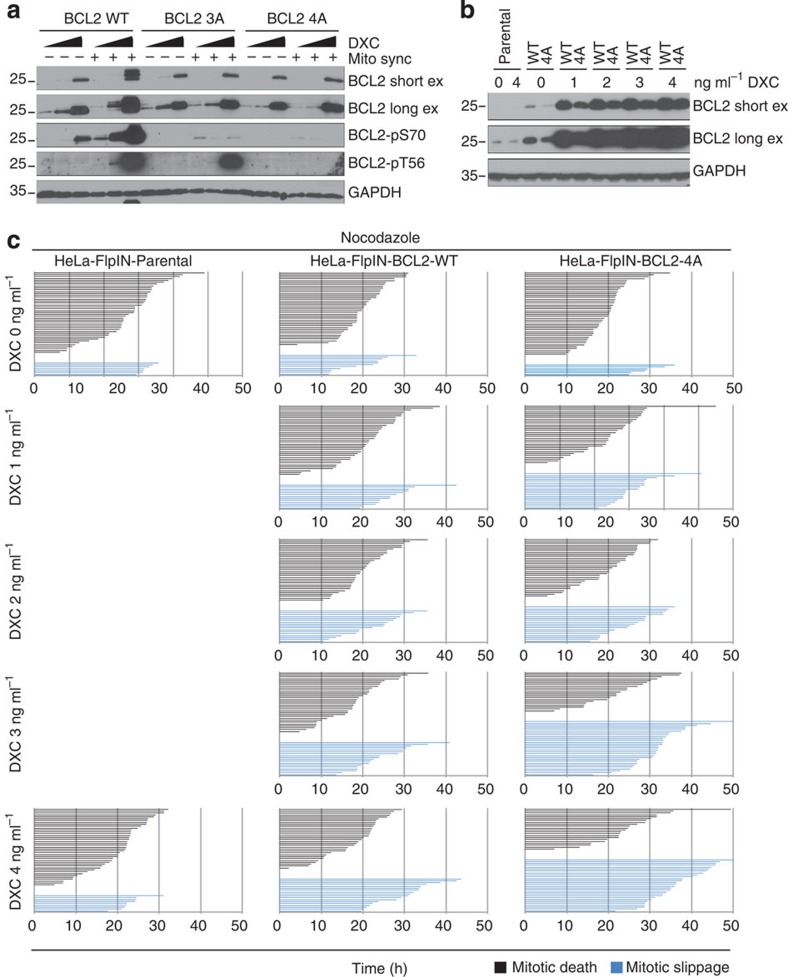
BCL2 phosphorylation in M barely inhibits its activity. (**a**) HeLa-FlpIN cells transgenic for BCL2-WT, BCL2-3A and BCL2-4 A were treated with 0, 4 and 200 ng ml^−1^ doxycyclin (DXC) for 24 h and were either left untreated (Mito sync −) or subjected to single thymidine arrest followed by release into Nocodazole followed by mitotic shake-off (Mito sync +). Lysates were analysed by immunoblotting using the indicated antibodies. For BCL2, a short and a long exposure are displayed. (**b**) HeLa-FlpIN-parental, BCL2-WT and BCL2-4A transgenic cells were treated with the indicated amounts of doxycyclin for 24 h, and subjected to either harvesting and immunoblot analysis or (**c**) time-lapse video microscopy on treatment with Nocodazole. Fate profiles of individual cells are displayed. Time in hours is indicated.

To directly test the impact of BCL2 phosphorylation at single cell resolution, we generated HeLa-FlpIN lines overexpressing either untagged-BCL2-3A- (carrying the triple mutation T69A, S70A and S87A) or BCL2-4A-carrying mutations of all known mitotic phospho-sites of BCL2 (T56A in addition). As on overexpression of BCL2 variants, the T56 phosphorylation in M became evident (Fig. 4a); we exploited our doxycycline (DXC)-regulated system to compare graded overexpression of BCL2-WT with BCL2-4A (Fig. 4b). On Nocodazole treatment, a slightly better protection of BCL2-4A over BCL2-WT-expressing cells was detected, judged based on the number of cells undergoing mitotic slippage (Fig. 4c). Similar results were obtained using BI2536 (Supplementary Fig. 2c,d). Together, these findings support the idea that CDK1/cyclin B-mediated phosphorylation of BCL2, and, by extrapolation, BCLX, may be required to render these cells susceptible to the effects of MCL1 degradation allowing BAK activation. They also demonstrate that phosphorylation does not significantly interfere with their ability to constrain BAX or BAK.

### NOXA is the rate-limiting BH3-only protein in M-arrest

As preventing phosphorylation of BCL2 only modestly improved its anti-apoptotic potential while ABT-737 treatment effectively synergized with MTA treatment in cell killing, we reasoned that CDK1/Cyclin B-mediated phosphorylation of BCL2 and/or BCLX followed by degradation of MCL1 may not suffice to effectively promote death in cells stalled in mitosis, arguing for an active contribution of BH3-only proteins in this process.

A systematic RNAi-based analysis targeting all BH3-only proteins reportedly expressed at protein and/or messenger RNA level in HeLa cells revealed dispensable or redundant roles for the BH3-only proteins BID, BAD, PUMA and BMF in this process (Supplementary Fig. 3).

The BH3-only protein BIM was heavily phosphorylated and subsequently degraded in mitotically arrested cells (Supplementary Fig. 4a). These observations are in line with its documented role as a substrate of mitotic kinases and target of the APC/C and/or SCF-betaTrcp^39^,^40^,^41^. However, in our hands, one BIM siRNA moderately depleting the protein had no impact on cell death, whereas a more complete depletion of BIM led to a small yet significant extension of the lifespan of mitotically arrested cells (Supplementary Fig. 4b–d). The use of either siRNAs led on the other hand to an extension of the duration of normal mitosis and the time required to slip, both correlating with the extent of protein depletion and highlighting a previously unrecognized function of BIM in mitosis and in mitotic slippage (Supplementary Fig. 4b–d).

**Figure 5:**
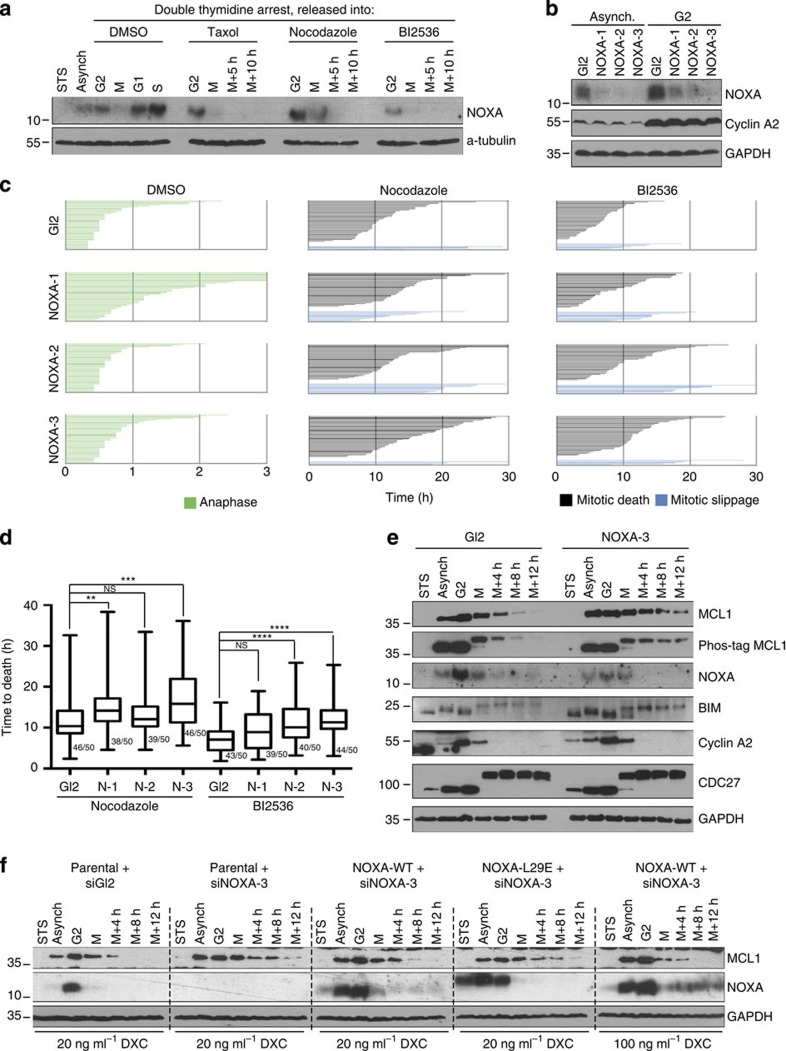
NOXA controls the mitotic lifespan by promoting MCL1 degradation. (**a**) HeLaS3 cells were synchronized and treated as described in Fig. 3 and processed for immunoblotting to evaluate NOXA expression. (**b**) Cells were transfected with the indicated siRNAs and either left untreated (Asynch.) or synchronized by double-thymidine block and harvested after the release in G2. Cells were analysed by immunoblotting (**c**) Fate profiles of individual HeLaS3-H2B–GFP cells subjected to treatment with DMSO, Nocodazole or BI2536 after transfection with the indicated siRNAs. Time in hours is indicated (**d**) Box (interquartile range) and whisker (min to max) plots showing the elapsed time (min) between nuclear envelope breakdown (NEBD) and cell death for individual cells traced in **c**. The fraction (X/50 events) of cells undergoing the fate of interest is indicated. **P<0.01; ***P<0.001; ****P<0.0001 using Mann–Whitney test. (**e**) HeLaS3 cells were transfected with the indicated siRNAs, synchronized by double-thymidine block, harvested after increasing times of mitotic arrest, triggered by Nocodazole and processed for immunoblot with the indicated antibodies. (**f**) Parental HeLa-FlpIN cells or transgenic for either NOXA-WT or NOXA-L29E were transfected with the indicated siRNAs, treated with Staurosporine (STS), left asynchronous (Async) or synchronized by double-thymidine block and released into medium containing Nocodozole and 20 or 100 ng ml^−1^ doxocycline (DXC). Cells were harvested in G2 or after increasing times of mitotic arrest and processed for immunoblot with the indicated antibodies. NS, not significant.

Most strikingly, however, we observed an accumulation of NOXA, the only selective MCL1 antagonist within the BH3-only subgroup in G2, that was followed by a rapid drop in M phase cells, the time when MCL1 levels start to decline (Fig. 5a,b), suggesting that the two phenomena might be linked. In line with a pro-death role of NOXA under these conditions, three different RNAi sequences targeting NOXA effectively delayed time to death in mitotically arrested cells (Fig. 5b–d). Of note, depletion of NOXA clearly increased MCL1 stability in M-arrested cells (Fig. 5e), accounting for the observed survival benefit and consistent with the notion that NOXA promotes proteasomal co-degradation of MCL1 on binding^42^. As MCL1 degradation in mitosis requires priming phosphorylation events^22^,^23^, we wondered whether NOXA depletion interfered with MCL1 phosphorylation. MCL1 displayed a marked mitotic shift on phos-tag gels both in the presence and in the absence of NOXA siRNA (Fig. 5e), suggesting that NOXA promotes MCL1 degradation downstream of its phosphorylation. Importantly, depletion of endogenous NOXA by targeting the 3́ untranslated region of its messenger RNA by the siRNA#3 allowed re-expression of untagged NOXA at varying expression levels, achieved by adding different concentrations of DXC, showing that MCL1 degradation can be rescued in a dose-dependent manner, arguing for the specificity of the NOXA siRNA phenotype (Fig. 5f). Furthermore, re-expression of NOXA carrying a point mutation in its BH3 domain (L29E) failed to abrogate MCL1 stabilization triggered by NOXA siRNA (Fig. 5f). Taken together our data suggest that NOXA contributes to MCL1 degradation by directly binding to it and possibly acting as a scaffold for a common E3 ligase in M-arrest.

**Figure 6:**
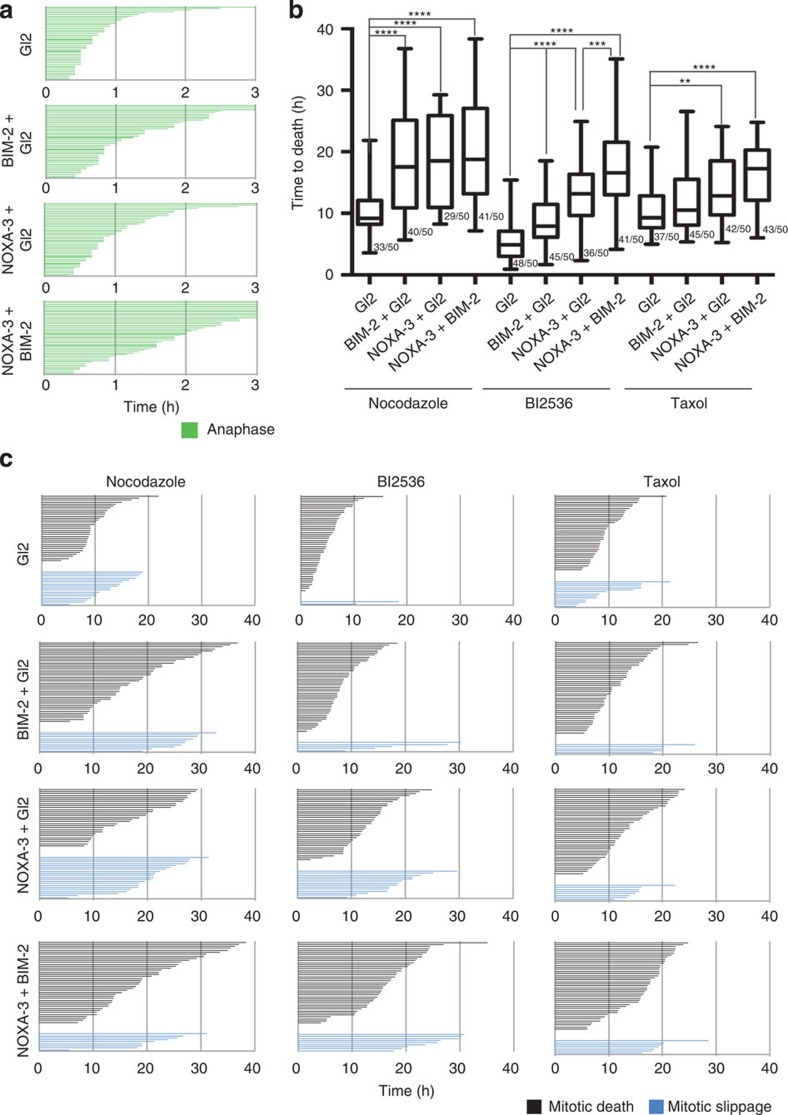
NOXA and BIM synergize in promoting mitotic cell death. (**a**) HeLaS3-H2B–GFP cells were transfected with a fixed amount of total siRNA using the indicated oligonucleotides and subjected to live cell imaging. Fate profiles of individual cells subjected DMSO treatment. Time is indicated in hours. (**b**) Box (interquartile range) and whisker (min to max) plots showing the elapsed time (h) between nuclear envelope breakdown (NEBD) and cell death for individual cells on the siRNA transfection in combination with Nocodazole, BI2536 or Taxol treatment. **P<0.01; ***P<0.001; ****P<0.0001 using Mann–Whitney test. The fraction (X/50 events) of cells undergoing the fate of interest is indicated. (**c**) Fate profiles of individual cells treated with the indicated drugs after transfection with the indicated siRNAs. Time in h is indicated.

As NOXA depletion also led to a relative increase in BIM levels (Fig. 5e), but BIM RNAi provided only limited protection (Supplementary Fig. 4c,d), we wondered if NOXA may be the driver and BIM the auxiliary BH3-only protein under these conditions that needs to be freed from sequestration by MCL1, or other pro-survival proteins, to execute apoptosis. To address this possibility, we performed RNAi co-depletion experiments in HeLa cells that supported our hypothesis that BIM and NOXA can cooperate in killing mitotically arrested cells (Fig. 6).

**Figure 7:**
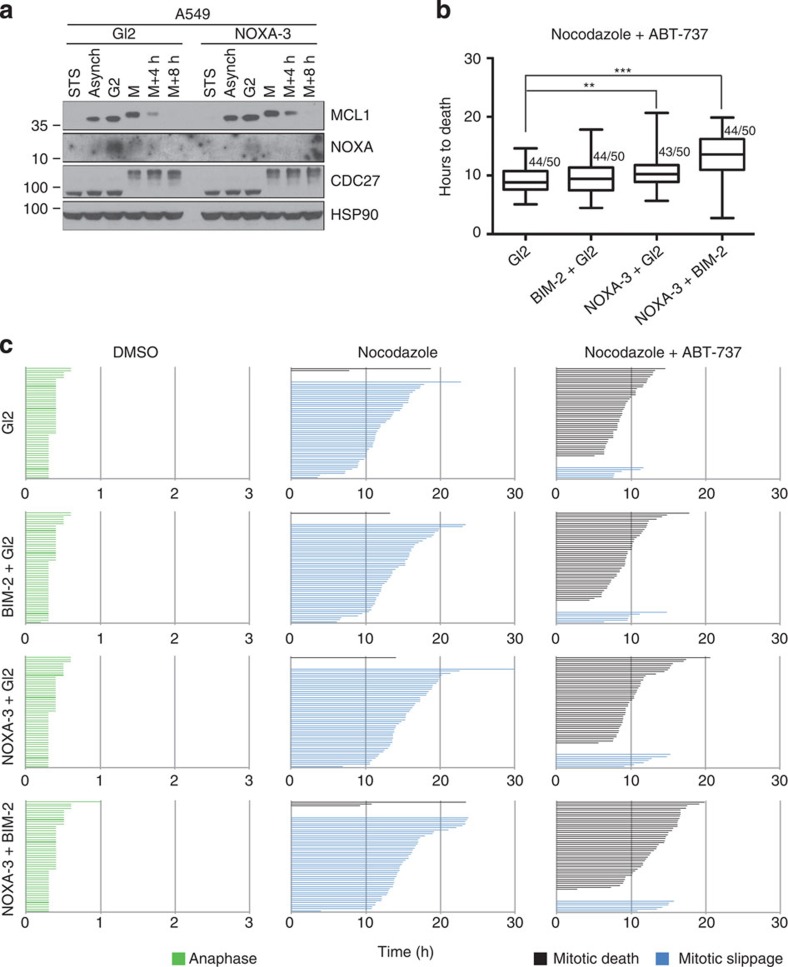
Noxa and Bim synergize in promoting mitotic death in A549 cells. (**a**) A549 cells were transfected with the indicated siRNAs, synchronized with double-thymidine block and released into medium containing Nocodazole. Cells were harvested after increasing times of mitotic arrest and processed for immunoblotting with the indicated antibodies. (**b**,**c**) A549-H2B-mRFP cells were transfected with the indicated siRNAs, synchronized, subjected to treatment with DMSO, Nocodazole or Nocodazole and ABT-737 and analysed by live cell imaging. (**b**) Box (interquartile range) and whisker (min to max) plots showing the elapsed time (h) between nuclear envelope breakdown (NEBD) and cell death for individual cells. **P<0.01; ***P<0.001 using Mann–Whitney test. The fraction (X/50 events) of cells undergoing the fate of interest is indicated. (**c**) Fate profiles of individual cells from time is indicated in hours.

**Figure 8:**
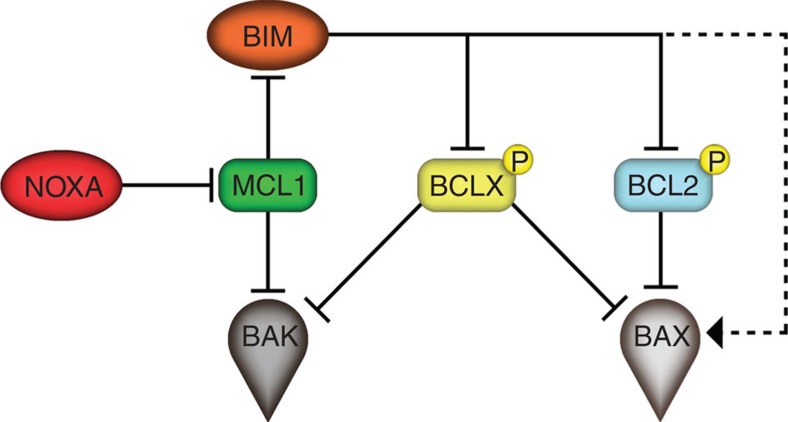
The M-death model. Proposed interrelation of BCL2 family proteins in apoptosis triggered on extended mitotic arrest. Despite their mitotic phosphorylation, BCL2 and BCLX retain their ability to inhibit BAX and BAK. On NOXA-driven MCL1 degradation BAK inhibition is abolished and increased amounts of BIM become available for either inhibiting BCL2 and BCLX or directly activating BAX.

To scrutinize the possibility that our observations might be limited to the HeLa cell system, we focused on A549 lung adenocarcinoma cells that have been defined as slippage prone due to a high BCLX activity that prevents cell death on extended M-arrest^24^. In those cells NOXA expression peaks also in G2 and shows a decay in the course of M-arrest, as in HeLa (Fig. 7a). When treated with Nocodazole, the majority of A549 cells underwent mitotic slippage, whereas co-treatment with ABT-737 forced them to undergo cell death during M-arrest (Fig. 7b,c). In this context, the NOXA depletion led to a significant extension to the time required for mitotic death, consistent with the observed stabilization of MCL1 (Fig. 7a–c). Of note, co-depletion with BIM further extended the time to cell death (Fig. 7b,c), corroborating our findings made in HeLa cells in support of a model, where the NOXA/MCL1/BIM axis drives cell death on extended M-arrest (Fig. 8).

Finally, we also employed HoxB8-immortalized mouse bone marrow-derived myeloid progenitor cells, generated from BH3-only protein knockout mice^43^ to investigate if the same BH3-only proteins may operate in mitotic cell death in murine cells. Loss of Bim or Noxa partially protected from cell death induction, while cells from double-mutant mice where more resistant to Nocodazole treatment (Supplementary Figs 5 and 6). While these experiments do not exclude that other BH3-only proteins may be engaged when Bim and Noxa are lacking, the experiments indicate that similar mechanisms execute cell death on stalled mitosis in mice.

## Discussion

Here we aimed to assign relative contributions to individual members of the BCL2 protein family in the process of apoptosis on forced M-arrest. Our data support a model of apoptosis in this paradigm of cell death that requires the BH3-only proteins NOXA and BIM as central players where the former is needed to effectively degrade MCL1 to allow BIM to act as auxiliary factor neutralizing remaining pro-survival family members, such as BCL2 or BCLX, or by directly promoting BAX activation (Fig. 8).

Our study was built on the assumption that apoptosis occurring during M-arrest must be a distinct cell death process, possessing unique features and differing in quality from apoptosis occurring in interphase as the majority of apoptotic regulators undergo post-translational modifications and transcription is absent during mitosis. Our data recapitulate some known phenomena and reconcile part of the existing controversies: first of all, mitochondrial apoptosis, controlled by the BCL2 family, mediates cell death on MTA treatment and ectopic expression of anti-apoptotic BCL2-family members, similar to caspase inhibition, prevents cell death and results in increased mitotic slippage (Fig. 1). Notably, in HeLa cells, this death appears to be mainly BAK driven (Fig. 2). This finding is in line with a rate-limiting role of its primary binding partner and antagonist, MCL1 (see below). A role of Caspase-8, a key component in a cell death-signalling complex dubbed the Ripoptosome, implicated in cell death paradigms that can associate with DNA-damage and subsequent cell cycle arrest^32^,^33^, however, was excluded by our study (Supplementary Fig. 1). Similarly, inhibition of RIP1 kinase, essential for necroptotic cell death^44^, had no impact on death in mitotically arrested cells (not shown). Second, we confirmed that BCL2 and BCLX inter-domain regions undergo mitotic phosphorylation (Fig. 3 and Supplementary Fig. 2). This has been reported by others but was interpreted in various ways assigning inhibitory as well as activating roles to mitotic phosphorylation events^38^,^39^,^45^,^46^. Importantly, the phosphorylation events that we scrutinized in our study are *bona fide* mitotic events as they do not vary quantitatively or qualitatively on increasing the time spent in mitosis and are immediately reverted on mitotic exit (Fig. 3 and Supplementary Fig. 2b). This suggests in turn that they can only alter the threshold required to trigger MOMP on other initiating changes, but they are not causative for life versus death decisions, as proposed by others^39^. Our data also reveal that BCL2 and BCLX activity is largely retained during mitosis despite their effective phosphorylation and highlights that the impact on protein function is minimal, contrasting other claims^47^. In line with two former seminal studies^22^,^23^, evidence presented here indicate that MCL1 mitotic degradation is substantially more critical for the induction of mitotic apoptosis. First, mitotic death in HeLa depends more on BAK than on BAX (Fig. 2), in line with evidence showing that MCL1 preferentially neutralizes BAK but not BAX^48^,^49^. Second, we detected both BAK and BAX activation on extended M-arrest using conformation-specific antibodies (Fig. 2). However, the inhibition of BCL2 and BCLX with ABT-737 resulted in enhanced activation of both BAK and BAX (Fig. 3). Taken together, the mitotic neutralization of MCL1 is the decisive event, leading to apoptosis on extended M-arrest.

The molecular machinery involved in MCL1 degradation received a lot of attention but it remains controversial and appears highly redundant, involving multiple ligases, including MULE, the APC/C-Cdc20 and/or SCF/FBW7 (refs 22, 23, 24). As the latter two E3s reportedly act on MCL1 in a phosphorylation-dependent manner and the residues involved partly overlap^21^, we used our FlpIN inducible system to conditionally overexpress phospho-defective mutants. Surprisingly though, none of the single (such as S159A or T163A) or double mutants that we tested resulted in MCL1 stabilization (not shown), suggesting that even the confirmed mitotic phospho-sites of MCL1 may act redundantly in triggering mitotic degradation together with other modifications. Regardless, NOXA depletion resulted in a clear mitotic stabilization of MCL1 (Fig. 5e,f).

While analysing NOXA protein expression in our synchronized samples, we noted that NOXA protein levels accumulate already in G2 (Figs 5 and 7). As this was true not only for the endogenous protein but also for ectopically expressed NOXA (Fig. 5f), we conclude that the above accumulation relies on a post-transcriptional/translational component that acts in G2 by either increasing NOXA translation or by stabilizing NOXA protein. Despite NOXA accumulation, MCL1 protein was clearly detectable in G2, suggesting that either only a fraction of NOXA may be bound to MCL1 or that the common E3 ligase is not yet active. The above-mentioned MCL1 (or possibly also NOXA) mitotic phosphorylation might then be critical for triggering co-degradation of both proteins, facilitating the assembly of a higher order protein complex that contains NOXA/MCL1 and one of its predicted E3 ligases. Given the impact that NOXA depletion has on MCL1 degradation, identifying the factors leading to NOXA accumulation in G2 may provide a new therapeutic angle for modulating the propensity of cells to undergo mitotic apoptosis, enhancing, therefore, the potency and specificity of anti-mitotic drugs such as MTAs.

While in HeLa cells, mitotic apoptosis can be accelerated by inhibition of BCL2 and BCLX, the combination treatment with an anti-mitotic drug together with ABT-737 can revert the slippage propensity of A549 cells, forcing them to die in a NOXA/BIM-dependent manner in the course of M-arrest. Our work not only strengthens the rational for combinatorial treatment of MTAs with BH3 mimetics in anticancer therapy, as already emphasized by others^50^,^51^, but also shows that the NOXA–MCL1–BIM axis is operational in different cell lines (Fig. 7). Although the universal relevance of our findings still awaits verification, our model is supported by data in two cancer cell lines of independent origin, HeLa and A549, that are characterized by high and low propensity for mitotic cell death and recognized as defective or functional in the p53 pathway, respectively. Hence, we suggest that our findings might be relevant for a variety of human tumour cell types and, based on our HoxB8 progenitor results (Supplementary Fig. 6), that the mechanism reported may also be conserved from mice to men.

As a note on the side, it is worth mentioning that we actually detected two qualitatively distinct phenotypes while modulating the mitochondrial pathway of apoptosis. When employing pan-caspase inhibition, we observed (1) increased slippage, associated with an extended time to death in those cells that eventually did die in the presence of QVD (Fig. 1b,c). BCL2 overexpression and BAK depletion led to a phenotype falling into the same category (Figs 1d and 2, respectively). These observations are in line with the notion that the apoptotic and the slippage networks act independently in competition with each other^17^,^18^,^52^. However, interfering with cell death at the level of BH3-only proteins NOXA (Fig. 5) or BIM (Supplementary Fig. 4), (2) significantly extended the time that cells required to die in mitosis without increasing the propensity of the cells to slip. In the case of BIM RNAi, we observed not only a lack of increase in cells slipping, but the cells that did slip also required more time for doing so (Supplementary Fig. 4). These observations are consistent with a positive contribution of the BH3-only protein BIM and possibly NOXA not only to mitotic apoptosis, but also to mitotic slippage. Collectively our data suggest that modulating different BCL2 family members involved in mitotic cell death can lead to distinct mitotic phenotypes that reflect either inhibition of MOMP alone (BCL2 overexpression and BAK depletion) or MOMP and mitotic slippage (BIM and NOXA depletion). Although not detected in our analysis, BAX/BAK double depletion has been shown to negatively impact mitotic slippage, proposing that defective mitochondrial fission, most likely via reduced BAX-modulated DRP1 activation, can impact on the propensity of cells to undergo slippage by indirectly affecting Cyclin B activity^53^. How exactly MOMP and mitochondrial dynamics are entangled particularly during mitosis remains to be a subject of future investigations.

## Methods

### Cell synchronization and drug treatment

Synchronization of HeLa cells was either done by a single 2 mM thymidine (Sigma-Aldrich, T1895) arrest for 24 h (Figs 1, 2a,b, 3d, 4a,c, 5c–e,g and 6 and Supplementary Figs 1c, 2b, 3C–E, 4C–D, 5A) or a double-thymidine arrest (22 h arrest followed after 9 h by a 17 h arrest (Figs 2d, 3a–c and 5a,f and Supplementary Figs 3a, 4a and 5b). After washout drugs were added: 1 μM Nocodazole (M1404, Sigma-Aldrich), 0.1 μM BI2536 (S1109, Selleck), 10 μM QVD (SML0063, Sigma-Aldrich), 0.5 μM reversine (BML-SC104, Enzo Life Sciences), 2.5 μM (in Fig. 7) or 1 μM (in all other Figures) ABT-737 (S1002, Selleck) were added. Dimethyl sulfoxide (D5879, Sigma-Aldrich) was used as solvent control. G2 time points were harvested after 8 h and the first mitotic time point (‘M') 11 h after release from thymidine arrest. All mitotic time points were harvested by shake-off. Doxycycline (D9891, Sigma-Aldrich) was used in the indicated concentrations. Staurosporine (S-9300, LC Laboratories) was used on asynchronous cells for 4 h at 1 μM.

### Generation of stable cell lines

The ‘HeLa-FlpIN-parental' strain was generated by transducing HeLa-FRT-H2B-mRED cells^34^ with pLib-tetR-KRAB-IRES-Blast viral supernatant, followed by selection with 8 μg ml^−1^ blasticidin (R210-01, Invitrogen) for 2 weeks. Untagged-BCL2 variants and NOXA were expressed by a pcDNA5/FRT/TO (Invitrogen) vector, whereas Strep-HA tagged CrmA was expressed by pTO-HA-Strep-GW-FRT vector^54^. BCL2 point mutations were obtained by Quickchange mutagenesis (Agilent). All constructs had been sequence verified. Stable integration into HeLa-FlpIN-parental was achieved by co-transfecting a total amount of 1.2 μg plasmid DNA in a 1:5 ratio (Invitrogen, expression vector:pOG44) in 100,000 cells, followed by 2 weeks of hygromycin B selection (300 μg ml^−1^ CP12.2, Roth). A549-H2B-mRFP were generated by transduction of A549 cells with a pLib-H2B-mRFP-ires-PURO lentiviral supernatant followed by selction with puromycin (1 μg ml^−1^)^55^.

### siRNA transfection

About 40 nM of siRNA were premixed with 2 μl ml^−1^ Oligofectamine (12252-011, Life Technologies) in Optimem (31985-054, Life Technologies) and incubated for 20 min at room temperature before transfection of the cells. Transfection took place 72 h before the start of life cell imaging or harvest for immunoblot/quantitative reverse transcription PCR analysis.

### Cell lysis and immunoblot

Cells were harvested either by trypsinization when asynchronous or in mitosis by selective shake-off. Samples were lysed in 50 mM Tris pH 8.0, 150 mM NaCl, 0.5% NP-40, 50 mM NaF, 1 mM Na_3_VO_4_, 1 mM PMSF, one tablet protease inhibitors (EDTA free, Roche) per 10 ml and 30 μg ml^−1^ DNaseI (Sigma-Aldrich). Protein concentration was measured by Bradford analysis (500-0006, Bio-rad).

Proteins were electro-blotted on AmershamTM HybondTM—ECL nitrocellulose membranes (GE Healthcare). For Phos-tag SDS–PAGE, 20 μM of Phos-tag (Phos-tagTM Acrylamide AAL-107, Wako Pure Chemical Industries, Ltd.) and 40 μM of MnCl_2_ (105927, Merck) were added to the poly-acrylamide solution and degassed for 10 min by vacuum centrifugation (SpeedVac SPD111V-230, Thermo Fisher Scientific).

### Time-lapse video microscopy

Following siRNA transfection or DXC treatment and a 24 h thymidine arrest, cells were treated with the respective drugs in a 12-well plate and imaged every 5 min by a screening microscope (Cell IQ SLF or Cell IQ MLF, Chip Man Technologies) with a × 10 objective. The time-lapse movies displayed in the bulk diagrams of Fig. 7 have been acquired with a × 10 objective on a TILL Photonics iMIC digital microscope equipped with a Hamamatsu OrcaFlash 4 camera. For analysis, 50 cells per treatment entering mitosis were randomly selected and duration of mitosis was manually assessed using Fiji.

### Statistical analysis

Statistical analysis was performed using Prism, Graphpad Software. The Mann–Whitney test was employed, with the exception of Supplementary Fig. 5, where a paired *t*-test was employed.

### Intracellular staining and flow cytometry

Intracellular staining was done according to ref. 56 using mouse anti BAX 6A7 (sc23959, Santa Cruz Biotechnology), mouse anti BAK AB1 (AM03, Calbiochem) and goat anti mouse IgG Alexa Fluor 647 (A-21236, Life Technologies). Briefly, cells were fixed in 2% paraformaldehyde for 30 min at 4 °C, incubated in 1% FCS, 1% saponin in PBS with 0.5 μg of primary antibodies for 30 min at 4 °C. Cells were washed with 1% FCS in PBS and stained with secondary antibody for 30 min at 4 °C. Cells were washed twice with 1% FCS in PBS. Flow cytometric analysis was performed using LSR-Fortessa cell analyzer. For Annexin V/propidium iodide or SubG1staining, undifferentiated HoxB8-immortalized mouse myeloid progenitor cells were treated with Nocodazole without prior synchronization. After washing with AnnexinV-binding buffer (10 mM Hepes, 140 mM NaCl and 2.5 mM CaCl_2_), cells were incubated with AnnexinV–FITC or AnnexinV–APC (1:50, BD Pharmingen, Heidelberg, Germany) and propidium iodide (1 mg ml^−1^, Sigma) for 20 min, followed by flow cytometry analysis^43^.

### Immunoblotting

The location of pre-stained molecular weight markers (peqGOLD protein Marker V, Peqlab #27-2210) on nitrocellulose membranes (Amersham Protan #10600004), is reported for each blot. One annotated uncropped scan for each individual antibody used throughout the main figures is reported in Supplementary Fig. 7. The antibody dilutions are reported in the Supplementary Methods section. Membranes were blocked in 5% (w/v) milk in PBST for 1 h and incubated with primary antibodies overnight. All primary antibodies were diluted into milk with the exception of NOXA that was diluted in BSA (both 5% w/v into PBST). For phospho-specific antibodies, TBST was used instead of PBST throughout the whole procedure. Immunoblots were developed using Western Bright ECL Spray (Advansta K-12049-D50) and either Agfa Curix (34YAX) or GE Healthcare high performance chemoluminescence (28906837) films.

## Author contributions

M.D.H. performed the experiments, analysed the data, contributed to writing and preparation of the figures; C.S. performed the experiments, generated and characterized the cell lines; S.K. and R.H. performed the experiments; S.G. and G.H. provided the reagents, analysed the data and edited the manuscript; A.V. designed the research, analysed the data and wrote the paper; L.L.F. performed the experiments, designed the research, analysed the data, wrote the paper and conceived the study.

## Additional information

**How to cite this article:** Haschka, M. D. *et al.* The NOXA–MCL1–BIM axis defines lifespan on extended mitotic arrest. *Nat. Commun.* 6:6891 doi: 10.1038/ncomms7891 (2015).

## Supplementary Material

Supplementary Information Supplementary Figures 1-7, Supplementary Methods and Supplementary References

Supplementary Movie 1 Related to Figure 1B. HeLaS3-H2B-GFP cells subjected to timelapse video microscopy following exposure to DMSO. Phase contrast and GFP fluorescence images have been overlaid. Image acquisition every 11 minutes 20 seconds.

Supplementary Movie 2 Related to Figure 1B. HeLaS3-H2B-GFP cells subjected to timelapse video microscopy following exposure to QVD. Phase contrast and GFP fluorescence images have been overlaid. Image acquisition every 11 minutes 20 seconds.

Supplementary Movie 3 Related to Figure 1B. HeLaS3-H2B-GFP cells subjected to timelapse video microscopy following exposure to Nocodazole. Phase contrast and GFP fluorescence images have been overlaid. Image acquisition every 11 minutes 20 seconds

Supplementary Movie 4 Related to Figure 1B. HeLaS3-H2B-GFP cells subjected to timelapse video microscopy following exposure to Nocodazole and QVD. Phase contrast and GFP fluorescence images have been overlaid. Image acquisition every 11 minutes 20 seconds.

Supplementary Movie 5 Related to Figure 1B. HeLaS3-H2B-GFP cells subjected to timelapse video microscopy following exposure to BI2536. Phase contrast and GFP fluorescence images have been overlaid. Image acquisition every 11 minutes 20 seconds.

Supplementary Movie 6 Related to Figure 1B. HeLaS3-H2B-GFP cells subjected to timelapse video microscopy following exposure to BI2536 plus QVD. Phase contrast and GFP fluorescence images have been overlaid. Image acquisition every 11 minutes 20 seconds.

## References

[b1] Jordan M. A. & Wilson L. Microtubules as a target for anticancer drugs. Nat. Rev. Cancer 4, 253–265 (2004). 15057285 10.1038/nrc1317

[b2] Argyriou A. A., Koltzenburg M., Polychronopoulos P., Papapetropoulos S. & Kalofonos H. P. Peripheral nerve damage associated with administration of taxanes in patients with cancer. Crit. Rev. Oncol. Hematol. 66, 218–228 (2008). 18329278 10.1016/j.critrevonc.2008.01.008

[b3] Kavallaris M. Microtubules and resistance to tubulin-binding agents. Nat. Rev. Cancer 10, 194–204 (2010). 20147901 10.1038/nrc2803

[b4] Jackson J. R., Patrick D. R., Dar M. M. & Huang P. S. Targeted anti-mitotic therapies: can we improve on tubulin agents? Nat. Rev. Cancer 7, 107–117 (2007). 17251917 10.1038/nrc2049

[b5] Chan K. S., Koh C. G. & Li H. Y. Mitosis-targeted anti-cancer therapies: where they stand. Cell Death Dis. 3, e411 (2012). 23076219 10.1038/cddis.2012.148 PMC3481136

[b6] Medema R. H., Lin C. C. & Yang J. C. Polo-like kinase 1 inhibitors and their potential role in anticancer therapy, with a focus on NSCLC. Clin. Cancer Res. 17, 6459–6466 (2011). 22003073 10.1158/1078-0432.CCR-11-0541

[b7] Janssen A. & Medema R. H. Mitosis as an anti-cancer target. Oncogene 30, 2799–2809 (2011). 21339734 10.1038/onc.2011.30

[b8] Kitagawa K. Too early to say, "no targeting of mitosis!". Nat. Rev. Clin. Oncol. 8, 444 (2011). 21587220 10.1038/nrclinonc.2010.228-c1

[b9] Komlodi-Pasztor E., Sackett D., Wilkerson J. & Fojo T. Mitosis is not a key target of microtubule agents in patient tumours. Nat. Rev. Clin. Oncol. 8, 244–250 (2011). 21283127 10.1038/nrclinonc.2010.228

[b10] Senovilla L. *et al.* An immunosurveillance mechanism controls cancer cell ploidy. Science 337, 1678–1684 (2012). 23019653 10.1126/science.1224922

[b11] Zitvogel L., Galluzzi L., Smyth M. J. & Kroemer G. Mechanism of action of conventional and targeted anticancer therapies: reinstating immunosurveillance. Immunity 39, 74–88 (2013). 23890065 10.1016/j.immuni.2013.06.014

[b12] Mitchison T. J. The proliferation rate paradox in antimitotic chemotherapy. Mol. Biol. Cell 23, 1–6 (2012). 22210845 10.1091/mbc.E10-04-0335 PMC3248889

[b13] Vitale I., Galluzzi L., Castedo M. & Kroemer G. Mitotic catastrophe: a mechanism for avoiding genomic instability. Nat. Rev. Mol. Cell Biol. 12, 385–392 (2011). 21527953 10.1038/nrm3115

[b14] Barille-Nion S., Bah N., Vequaud E. & Juin P. Regulation of cancer cell survival by BCL2 family members on prolonged mitotic arrest: opportunities for anticancer therapy. Anticancer Res. 32, 4225–4233 (2012). 23060542

[b15] Musacchio A. & Salmon E. D. The spindle-assembly checkpoint in space and time. Nat. Rev. Mol. Cell Biol. 8, 379–393 (2007). 17426725 10.1038/nrm2163

[b16] Pines J. Cubism and the cell cycle: the many faces of the APC/C. Nat. Rev. Mol. Cell Biol. 12, 427–438 (2011). 21633387 10.1038/nrm3132

[b17] Gascoigne K. E. & Taylor S. S. Cancer cells display profound intra- and interline variation following prolonged exposure to antimitotic drugs. Cancer Cell 14, 111–122 (2008). 18656424 10.1016/j.ccr.2008.07.002

[b18] Huang H. C., Mitchison T. J. & Shi J. Stochastic competition between mechanistically independent slippage and death pathways determines cell fate during mitotic arrest. PLoS ONE 5, e15724 (2010). 21203573 10.1371/journal.pone.0015724 PMC3006339

[b19] Huang H. C., Shi J., Orth J. D. & Mitchison T. J. Evidence that mitotic exit is a better cancer therapeutic target than spindle assembly. Cancer Cell 16, 347–358 (2009). 19800579 10.1016/j.ccr.2009.08.020 PMC2758291

[b20] Ma H. T., Chan Y. Y., Chen X., On K. F. & Poon R. Y. Depletion of p31comet protein promotes sensitivity to antimitotic drugs. J. Biol. Chem. 287, 21561–21569 (2012). 22544748 10.1074/jbc.M112.364356 PMC3375577

[b21] Topham C. H. & Taylor S. S. Mitosis and apoptosis: how is the balance set? Curr. Opin. Cell Biol. 25, 780–785 (2013). 23890995 10.1016/j.ceb.2013.07.003

[b22] Harley M. E., Allan L. A., Sanderson H. S. & Clarke P. R. Phosphorylation of Mcl-1 by CDK1-cyclin B1 initiates its Cdc20-dependent destruction during mitotic arrest. EMBO J. 29, 2407–2420 (2010). 20526282 10.1038/emboj.2010.112 PMC2910263

[b23] Wertz I. E. *et al.* Sensitivity to antitubulin chemotherapeutics is regulated by MCL1 and FBW7. Nature 471, 110–114 (2011). 21368834 10.1038/nature09779

[b24] Shi J., Zhou Y., Huang H. C. & Mitchison T. J. Navitoclax (ABT-263) accelerates apoptosis during drug-induced mitotic arrest by antagonizing Bcl-xL. Cancer Res. 71, 4518–4526 (2011). 21546570 10.1158/0008-5472.CAN-10-4336 PMC3129452

[b25] Wang P. *et al.* Phosphorylation of the proapoptotic BH3-only protein bid primes mitochondria for apoptosis during mitotic arrest. Cell Rep. 7, 661–671 (2014). 24767991 10.1016/j.celrep.2014.03.050 PMC4022835

[b26] Kutuk O. & Letai A. Displacement of Bim by Bmf and Puma rather than increase in Bim level mediates paclitaxel-induced apoptosis in breast cancer cells. Cell. Death Differ. 17, 1624–1635 (2010). 20431602 10.1038/cdd.2010.41 PMC2914832

[b27] Miller A. V. *et al.* Paclitaxel-induced apoptosis is BAK-dependent, but BAX and BIM-independent in breast tumour. PLoS ONE 8, e60685 (2013). 23577147 10.1371/journal.pone.0060685 PMC3618047

[b28] Collin P., Nashchekina O., Walker R. & Pines J. The spindle assembly checkpoint works like a rheostat rather than a toggle switch. Nat. Cell Biol. 15, 1378–1385 (2013). 24096242 10.1038/ncb2855 PMC3836401

[b29] Lenart P. *et al.* The small-molecule inhibitor BI 2536 reveals novel insights into mitotic roles of polo-like kinase 1. Curr. Biol. 17, 304–315 (2007). 17291761 10.1016/j.cub.2006.12.046

[b30] Sanchez-Perez T., Medema R. H. & Lopez-Rivas A. Delaying mitotic exit downregulates FLIP expression and strongly sensitizes tumour cells to TRAIL. Oncogene 34, 661–669 (2014). 24488010 10.1038/onc.2013.601

[b31] Sanchez-Perez T., Ortiz-Ferron G. & Lopez-Rivas A. Mitotic arrest and JNK-induced proteasomal degradation of FLIP and Mcl-1 are key events in the sensitization of breast tumour cells to TRAIL by antimicrotubule agents. Cell. Death Differ. 17, 883–894 (2010). 19942932 10.1038/cdd.2009.176

[b32] Tenev T. *et al.* The Ripoptosome, a signaling platform that assembles in response to genotoxic stress and loss of IAPs. Mol. Cell 43, 432–448 (2011). 21737329 10.1016/j.molcel.2011.06.006

[b33] Feoktistova M. *et al.* cIAPs block Ripoptosome formation, a RIP1/caspase-8 containing intracellular cell death complex differentially regulated by cFLIP isoforms. Mol. Cell 43, 449–463 (2011). 21737330 10.1016/j.molcel.2011.06.011 PMC3163271

[b34] Klebig C., Korinth D. & Meraldi P. Bub1 regulates chromosome segregation in a kinetochore-independent manner. J. Cell Biol. 185, 841–858 (2009). 19487456 10.1083/jcb.200902128 PMC2711590

[b35] Tait S. W. & Green D. R. Mitochondrial regulation of cell death. Cold Spring Harb. Perspect. Biol. 5, a008706 (2013). 24003207 10.1101/cshperspect.a008706 PMC3753705

[b36] Czabotar P. E., Lessene G., Strasser A. & Adams J. M. Control of apoptosis by the BCL-2 protein family: implications for physiology and therapy. Nat. Rev. Mol. Cell Biol. 15, 49–63 (2014). 24355989 10.1038/nrm3722

[b37] Chin G. M. & Herbst R. Induction of apoptosis by monastrol, an inhibitor of the mitotic kinesin Eg5, is independent of the spindle checkpoint. Mol. Cancer Ther. 5, 2580–2591 (2006). 17041103 10.1158/1535-7163.MCT-06-0201

[b38] Deng X., Gao F., Flagg T. & May W. S. Jr. Mono- and multisite phosphorylation enhances Bcl2's antiapoptotic function and inhibition of cell cycle entry functions. Proc. Natl Acad. Sci. USA 101, 153–158 (2004). 14660795 10.1073/pnas.2533920100 PMC314154

[b39] Terrano D. T., Upreti M. & Chambers T. C. Cyclin-dependent kinase 1-mediated Bcl-xL/Bcl-2 phosphorylation acts as a functional link coupling mitotic arrest and apoptosis. Mol. Cell Biol. 30, 640–656 (2010). 19917720 10.1128/MCB.00882-09 PMC2812246

[b40] Mac Fhearraigh S. & Mc Gee M. M. Cyclin B1 interacts with the BH3-only protein Bim and mediates its phosphorylation by Cdk1 during mitosis. Cell Cycle 10, 3886–3896 (2011). 22071694 10.4161/cc.10.22.18020

[b41] Moustafa-Kamal M., Gamache I., Lu Y., Li S. & Teodoro J. G. BimEL is phosphorylated at mitosis by Aurora A and targeted for degradation by betaTrCP1. Cell. Death Differ. 20, 1393–1403 (2013). 23912711 10.1038/cdd.2013.93 PMC3770328

[b42] Czabotar P. E. *et al.* Structural insights into the degradation of Mcl-1 induced by BH3 domains. Proc. Natl Acad. Sci. USA 104, 6217–6222 (2007). 17389404 10.1073/pnas.0701297104 PMC1851040

[b43] Kirschnek S. *et al.* Molecular analysis of neutrophil spontaneous apoptosis reveals a strong role for the pro-apoptotic BH3-only protein Noxa. Cell. Death Differ. 18, 1805–1814 (2011). 21660046 10.1038/cdd.2011.69 PMC3190116

[b44] Degterev A. *et al.* Identification of RIP1 kinase as a specific cellular target of necrostatins. Nat. Chem. Biol. 4, 313–321 (2008). 18408713 10.1038/nchembio.83 PMC5434866

[b45] Dai H. *et al.* Contribution of Bcl-2 phosphorylation to Bak binding and drug resistance. Cancer Res. 73, 6998–7008 (2013). 24097825 10.1158/0008-5472.CAN-13-0940 PMC3910374

[b46] Choi H. J. & Zhu B. T. Role of cyclin B1/Cdc2 in mediating Bcl-XL phosphorylation and apoptotic cell death following nocodazole-induced mitotic arrest. Mol. Carcinog. 53, 125–137 (2014). 22949227 10.1002/mc.21956

[b47] Eichhorn J. M., Sakurikar N., Alford S. E., Chu R. & Chambers T. C. Critical role of anti-apoptotic Bcl-2 protein phosphorylation in mitotic death. Cell Death Dis. 4, e834 (2013). 24091677 10.1038/cddis.2013.360 PMC3824670

[b48] Dunkle A., Dzhagalov I. & He Y. W. Mcl-1 promotes survival of thymocytes by inhibition of Bak in a pathway separate from Bcl-2. Cell. Death Differ. 17, 994–1002 (2010). 20057504 10.1038/cdd.2009.201 PMC2866813

[b49] Willis S. N. *et al.* Proapoptotic Bak is sequestered by Mcl-1 and Bcl-xL, but not Bcl-2, until displaced by BH3-only proteins. Genes Dev. 19, 1294–1305 (2005). 15901672 10.1101/gad.1304105 PMC1142553

[b50] Han L. Y., Kipps E. & Kaye S. B. Current treatment and clinical trials in ovarian cancer. Expert Opin. Investig. Drugs 19, 521–534 (2010). 10.1517/13543781003647966 20367193

[b51] Oakes S. R. *et al.* Sensitization of BCL-2-expressing breast tumours to chemotherapy by the BH3 mimetic ABT-737. Proc. Natl Acad. Sci. USA 109, 2766–2771 (2012). 21768359 10.1073/pnas.1104778108 PMC3286989

[b52] Matson D. R. & Stukenberg P. T. Spindle poisons and cell fate: a tale of two pathways. Mol. Interv. 11, 141–150 (2011). 21540474 10.1124/mi.11.2.12 PMC3086919

[b53] Diaz-Martinez L. A. *et al.* Genome-wide siRNA screen reveals coupling between mitotic apoptosis and adaptation. EMBO J. 33, 1960–1976 (2014). 25024437 10.15252/embj.201487826 PMC4195789

[b54] Glatter T., Wepf A., Aebersold R. & Gstaiger M. An integrated workflow for charting the human interaction proteome: insights into the PP2A system. Mol. Syst. Biol. 5, 237 (2009). 19156129 10.1038/msb.2008.75 PMC2644174

[b55] Sigl R., Ploner C., Shivalingaiah G., Kofler R. & Geley S. Development of a multipurpose GATEWAY-based lentiviral tetracycline-regulated conditional RNAi system (GLTR). PLoS ONE 9, e97764 (2014). 24841113 10.1371/journal.pone.0097764 PMC4026376

[b56] Kepp O., Rajalingam K., Kimmig S. & Rudel T. Bak and Bax are non-redundant during infection- and DNA damage-induced apoptosis. EMBO J. 26, 825–834 (2007). 17235284 10.1038/sj.emboj.7601533 PMC1794390

