# Supplementary material for: The NOXA–MCL1–BIM axis defines lifespan on extended mitotic arrest
Source: Nat Commun. 2015 Apr 29;6:6891. doi: 10.1038/ncomms7891 (PMC4423218; doi:10.1038/ncomms7891)
Supplement: Supplementary Information — Supplementary Figures 1-7, Supplementary Methods and Supplementary References [file ncomms7891-s1.pdf]

Supplementary Figures

Haschka *et al*., Supplementary Figure 1

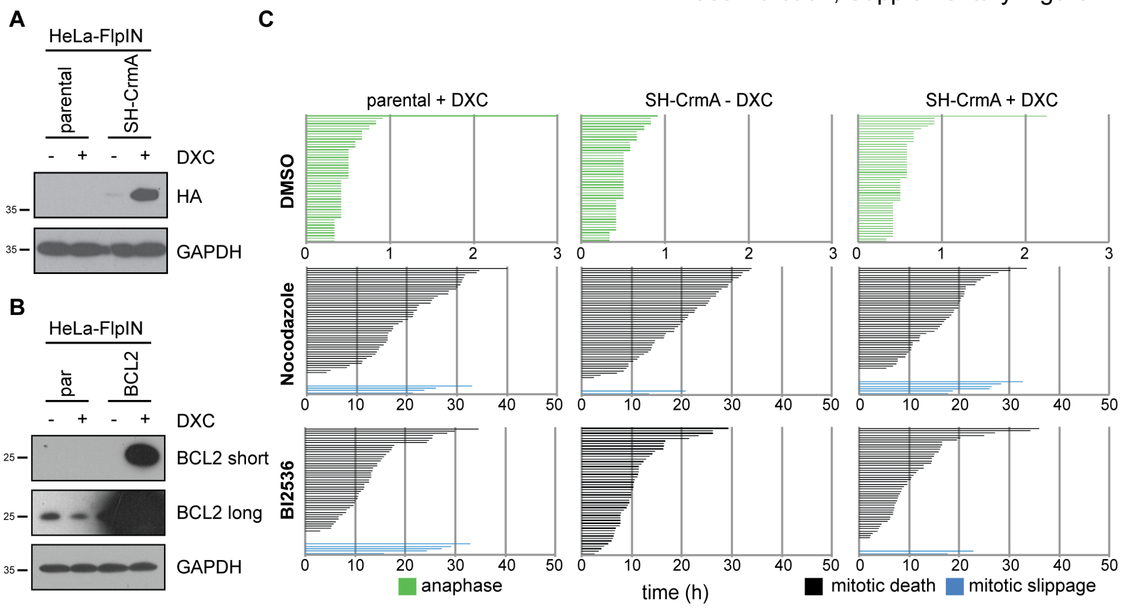

Supplementary Figure 1 CrmA overexpression does not affect mitotic apoptosis in HeLa-FlpIN cells, related to Figure 1. (A and B). Parental cells or cells in which the indicated construct was stably integrated were either left untreated or treated with Doxycycline 200 ng/ml (DXC) for 24 h. Immunoblotting with the indicated antibodies was performed. **(C)** Fate profiles of HeLa-FlpIN cells subjected to treatment with DMSO, Nocodazole or BI2536. Time in hours is indicated. Parental cells were pre-treated with 200 ng/ml Doxycycline for 24 h, whereas SH-epitope-tagged CrmA transgenic cells were either left untreated or pre-treated with 200 ng/ml Doxycycline for 24 h to induce the transgene.

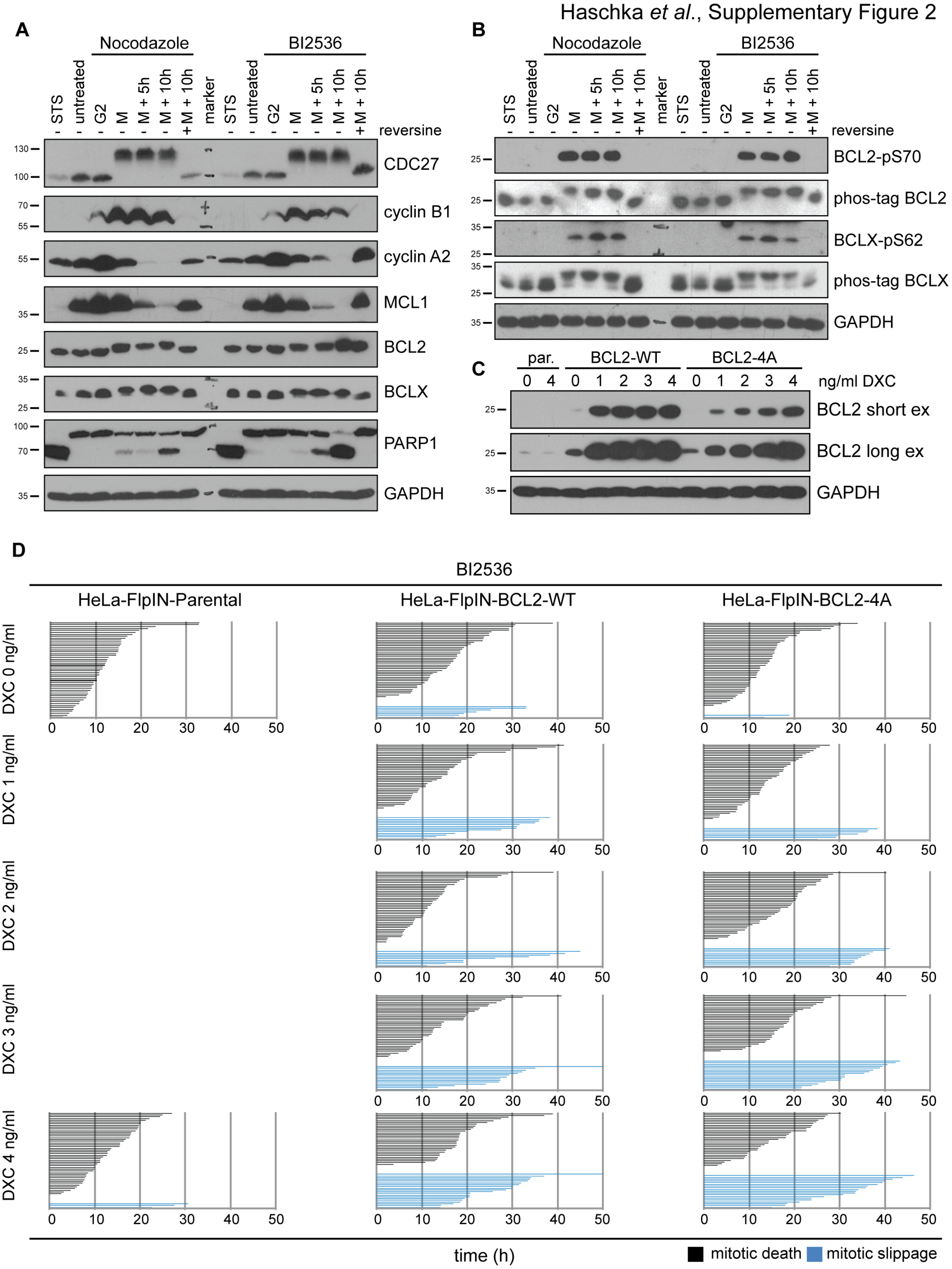

Supplementary Figure 2 BCL2 remains active, despite extensive phosphorylation, related to Figure 4. (A) Cells were asynchronous and left untreated and asynchronous (Asynch), treated with staurosporine (STS) or synchronized by double-thymidine block and released from arrest into Nocodazole or BI2536-containing media. Cells were harvested in G2, early M phase or following increasing time of M arrest. Reversine was added to trigger mitotic slippage. Cells were harvested, lysed and analyzed by immunoblotting. (**B**) Cells were

treated and processed as described in (A) and immunoblotting with the indicated antibodies was performed either on normal SDS PAGE or on phos-tag gels. **(C)** HeLa-FlpIN parental, BCL2-WT and BCL2-4A transgenic were treated with the indicated amounts of doxycyclin for 24 h, and subjected to either harvesting and immunoblot analysis or **(D)** time-lapse video microscopy upon treatment with BI2536**.** Fate profiles of individual cells are displayed. Time in hours is indicated.

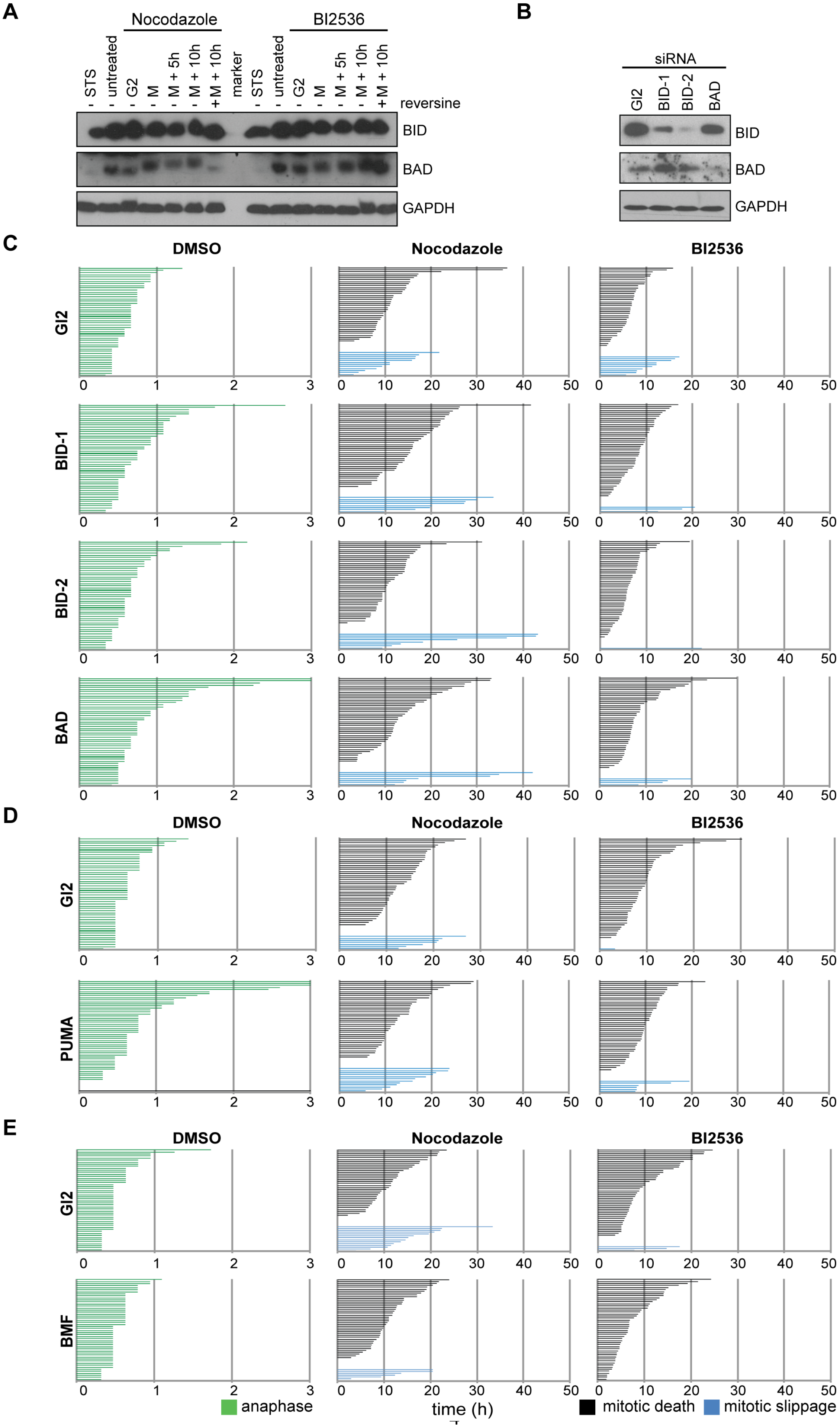

A
Nocodazole
BI2536
- STS
- untreated
- G2
- M
- M + 5h
- M + 10h
+ M + 10h
marker
- STS
- untreated
- G2
- M
- M + 5h
- M + 10h
+ M + 10h
reversine
BID
BAD
GAPDH
B
siRNA
Gl2
BID-1
BID-2
BAD
BID
BAD
GAPDH
C
DMSO
Nocodazole
BI2536
Gl2
BID-1
BID-2
BAD
D
DMSO
Nocodazole
BI2536
Gl2
PUMA
E
DMSO
Nocodazole
BI2536
Gl2
BMF
0 1 2 3
0 10 20 30 40 50
anaphase
time (h)
mitotic death
mitotic slippage

Supplementary Figure 3 BID, BAD, PUMA and BMF depletions have no impact on mitotic apoptosis, related to Figure 5. (A) HeLaS3 cells were synchronized and treated as described in Figure 3 and processed for immunoblot with the indicated antibodies. **(B)** HeLaS3-H2B-GFP cells were transfected with siRNAs, a fraction of the cells were harvested and analyzed by immunoblotting with the indicated antibodies, while the remaining cells were re-seeded and subjected to live-cell imaging: **(C - E)** fate profiles of individual cells subjected to DMSO, Nocodazole or BI2536 in combination with transfection with the indicated siRNAs. Time in hours is indicated.

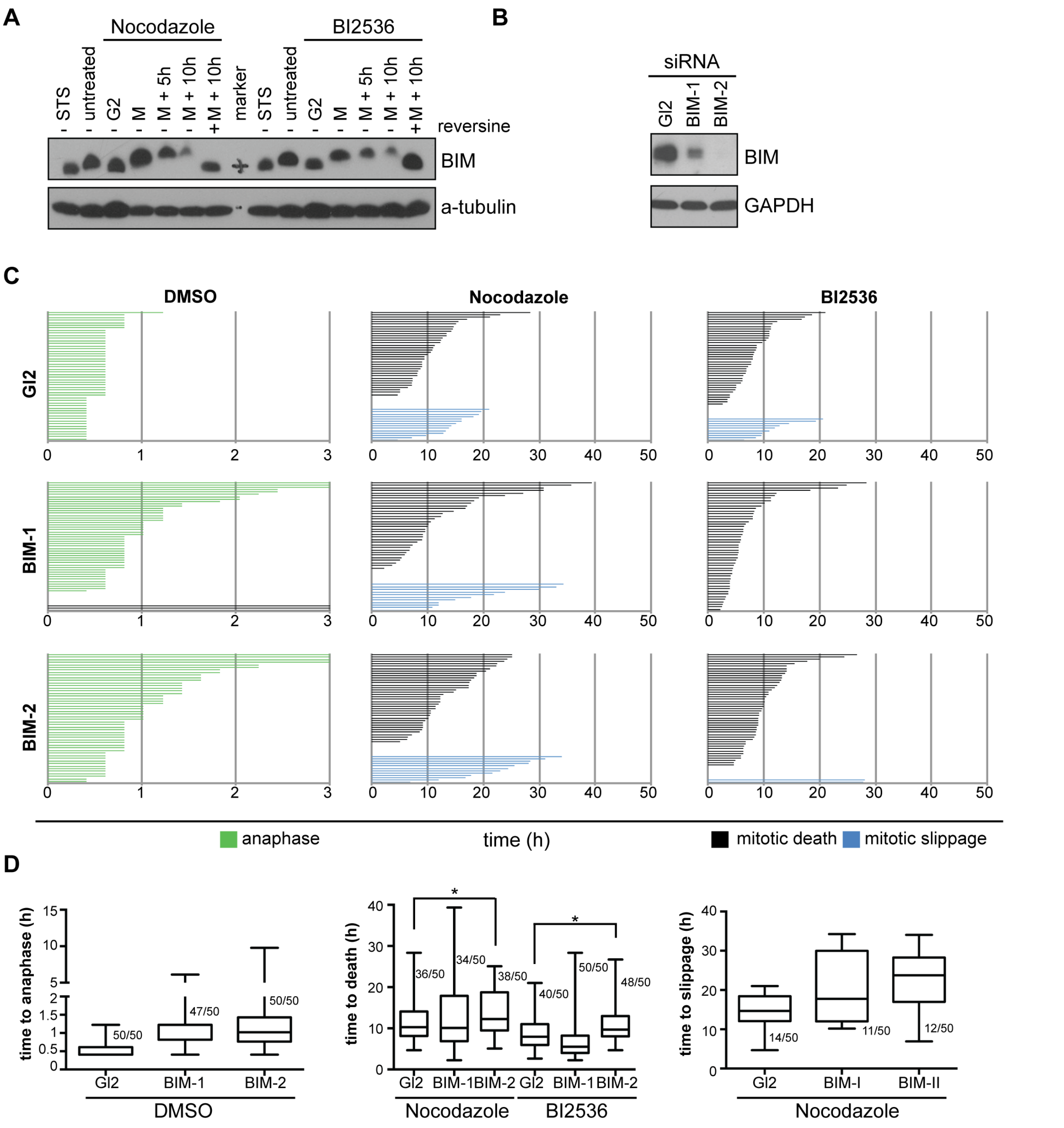

Supplementary Figure 4 BIM depletion delays both mitotic apoptosis and mitotic slippage, related to Figure 5. (A) HeLaS3 cells were synchronized and treated as described in Figure S2A-B and processed for immunoblot with the indicated antibodies. **(B-C)** HeLaS3-H2B-GFP cells were transfected with siRNAs, a fraction of the cells were harvested and analyzed by immunoblotting with the indicated antibodies, while the remaining cells were re-seeded and subjected to live cell imaging (C)**.** Fate profiles of individual cells subjected to DMSO, Nocodazole or BI2536 in combination with transfection with the indicated siRNAs. Time in hours is indicated. **(E)** Box (interquartile range) and whisker (min to max) plots showing the elapsed time (h) between NEBD and the indicated fate for

individual cells after treatment. The fraction (X/50 events) of cells undergoing the fate of interest is indicated.

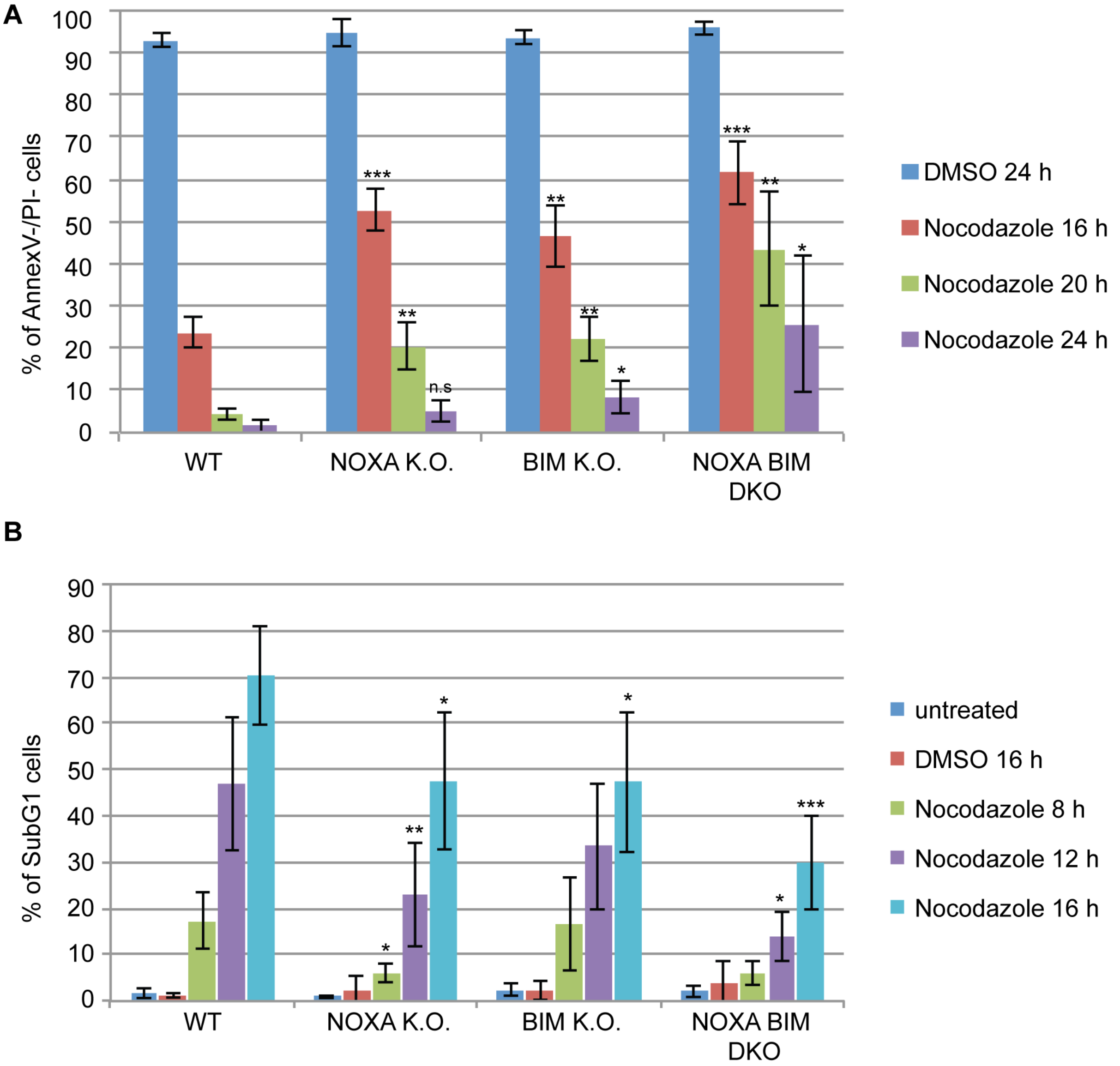

Supplementary Figure 5 Noxa and Bim synergize in promoting mitotic death in immortalized mouse bone marrow-derived myeloid progenitors, related to Figure 7. (**A**) AnnexinV/PI staining of undifferentiated progenitors was performed following increasing times of Nocodazole treatment for the indicated genotypes. The average percentage of double-negative cells ± SD is displayed (N=5). **(B)** Nicoletti-staining of cells treated as in (A), average percentage of SubG1 cells ± SD is displayed, N=3. *: $P < 0.05$; **: $P < 0.01$; ***: $P < 0.001$.

Haschka *et al.*, Supplementary Figure 6

A

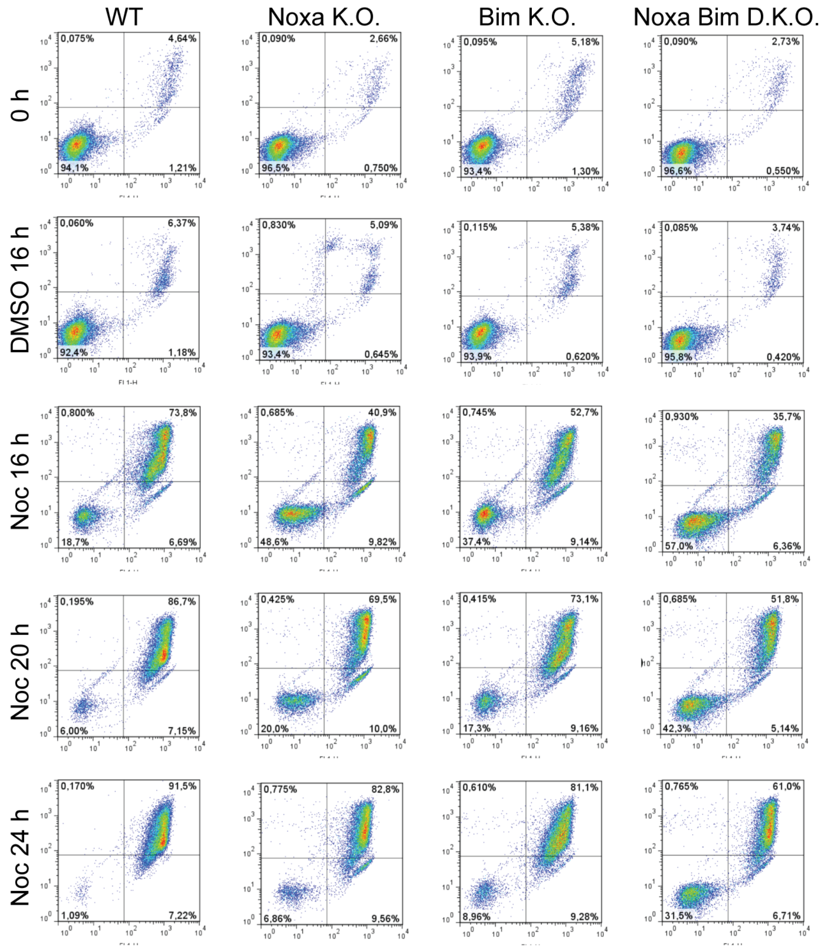

B

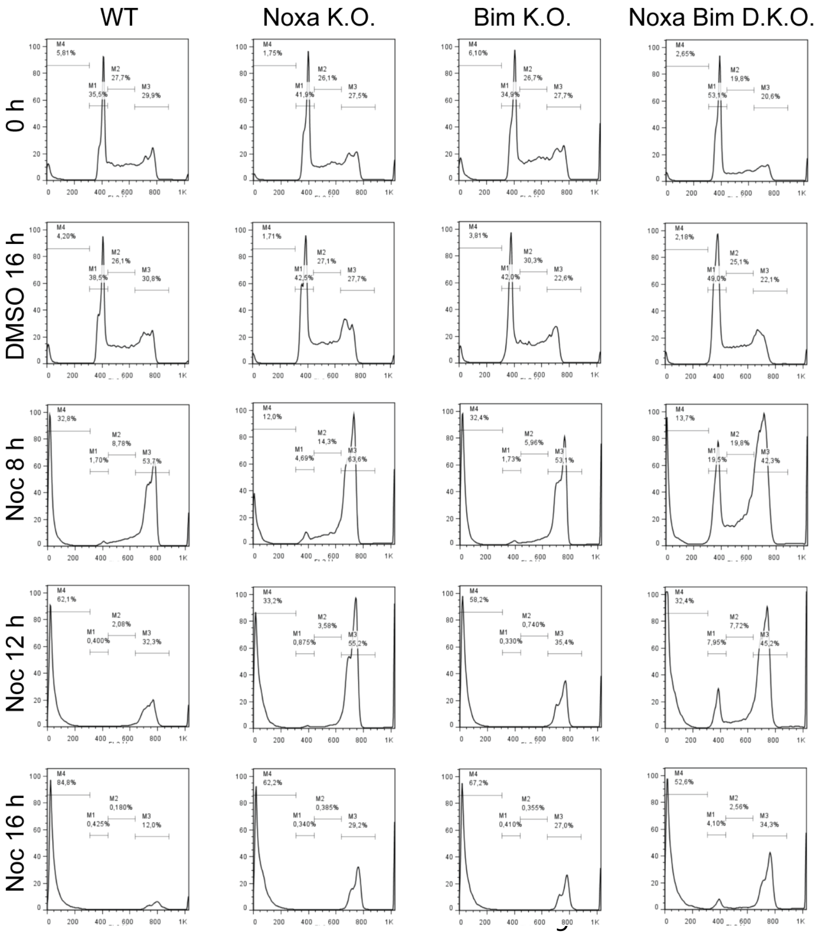

Supplementary Figure 6 Representative FACS plots, related to Figure S5. (A) AnnexinV/PI staining of undifferentiated progenitors was performed following increasing times of Nocodazole treatment for the indicated genotypes. Data obtained in one representative experiment are displayed. X=AnnexinV, Y=PI fluorescence. **(B)** Nicoletti stained progenitors following increasing times of Nocodazole treatment for the indicated genotypes. X=PI fluorescence, Y=cell count. Data obtained in one representative experiment are displayed.

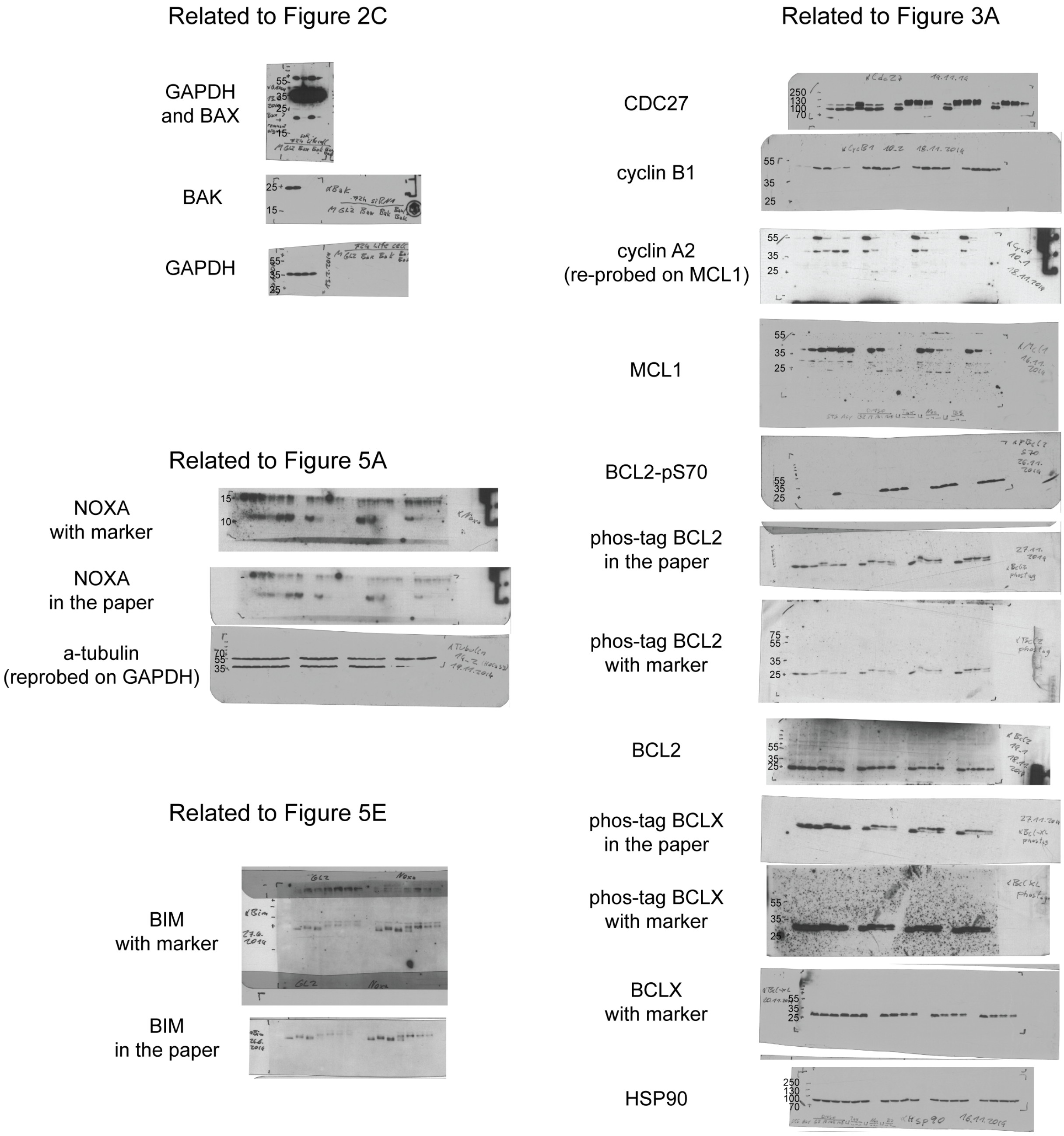

Supplementary Figure 7 Examples of uncropped scans of immunoblots.

Supplementary Methods

Cell culture. The cervix carcinoma cell line HeLaS3 was a gift of E. Nigg (University of Basel), HeLaS3 ± H2B-GFP [1] and HeLa H2B-mRed with an FRT-site for Flp-In [2] recombination were previously described. A549 lung adenocarcinoma cell line was a gift of P. Jennings (University of Innsbruck). All human cell lines were cultured in DMEM (PAA laboratories, E15-009 or Sigma-Aldrich, D5671) supplemented with 10% fetal bovine serum (FBS, PAA laboratories, A15-151), 1% L-glutamine (PAA laboratories, M11-004), 100 U/ml penicillin and 100 μg/ml streptomycin (PAA laboratories, P11-010). Cells were incubated at 37 °C with 5% CO2. HoxB8-immortalized mouse myeloid progenitor cells were cultured as described previously [3].

siRNA mediated protein depletion. The following siRNA duplexes were used: GL2: CGUACGCGGAAUACUUCGATT, BAX: GGUGCCGGAACUGAUCAGA, NOXA-1: CUUCCGGCAGAAACUUCUG, NOXA-2: GCAAGAACGCUCAACCGAGTT, NOXA-3: GGUGCACGUUUCAUCAAUUTT, BIM-1: UGAUGUAAGUUCUGAGUGUG, BIM-2: GGAGACGAGUUUAACGCUUA, BID-2: GAAUAGAGGCAGAUUCUGATT, BAD: AAGAAGGGACUUCCUCGCCCGTT, PUMA: UCUCAUCAUGGGACUCCUG and BMF: CACCGGCUUCAUGUGCAGCA, were all from Mycrosynth. BID-1: EHU157511 (Sigma-Aldrich), BAK: M-003305-02-0005 (Dharmacon).

Antibodies. The following antibodies were used for detection of proteins by chemoluminescence (Advansta, K-12049-D50): mouse anti CDC27 (clone 35/CDC27, 1:300), mouse anti Cyclin A2 (clone E23, 1:250), mouse anti Cyclin B1 (clone V152, 1:100), mouse anti Tubulin (Sigma-Aldrich, T6199, 0.5 µg/ml), rabbit anti PARP1 (Cell Signaling, #9542, 1:1000), anti rabbit BAX (Cell Signaling, #2772, 1:1000), anti rabbit BAK (Cell Signaling, #3814, 1:1000), mouse anti BCL2 (clone S100, 1 µg/ml), rabbit anti BCLX (Cell Signaling, #2764, 1:1000), rabbit anti MCL1 (Santa Cruz Biotechnology, sc-819, 0.4 µg/ml), rat anti BIM (Enzo Life Sciences, ALX-804-527-C100, 1 µg/ml), rat anti BID (clone 8C3, 1:1000), rabbit anti BAD (Cell Signaling, cat. N. #9292, 1:750), rabbit anti GAPDH (Cell Signaling, 2118, 1:5000), mouse anti NOXA (Abcam, ab13654, 1 µg/ml), rabbit anti BCL2-pS70 (Cell Signaling, #2827, 1:1000), rabbit anti BCL2-pT56 (Cell Signaling, #2875, 1:1000), anti BCLX-pS62 (Abcam, 30655, 1 µg/ml), mouse anti HA.11 (Covance, MMS-101P, 1:1000), goat anti rabbit Ig/HRP (Dako, P0448, 25 ng/ml), rabbit anti mouse Ig/HRP (Dako, P0161, 0.13 µg/ml), rabbit anti rat IgG heavy chain/HRP (Jackson ImmunoResearch, discontinued, 1:10000)

Supplemental References

1. Sillje HH, Nagel S, Korner R, Nigg EA. HURP is a Ran-importin beta-regulated protein that stabilizes kinetochore microtubules in the vicinity of chromosomes. *Curr Biol* 2006, **16**(8)**:** 731-742.

2. Klebig C, Korinth D, Meraldi P. Bub1 regulates chromosome segregation in a kinetochore-independent manner. *J Cell Biol* 2009, **185**(5)**:** 841-858.

3. Kirschnek S, Vier J, Gautam S, Frankenberg T, Rangelova S, Eitz-Ferrer P*, et al.* Molecular analysis of neutrophil spontaneous apoptosis reveals a strong role for the pro-apoptotic BH3-only protein Noxa. *Cell death and differentiation* 2011, **18**(11)**:** 1805-1814.

4. Bock FJ, Krumschnabel G, Manzl C, Peintner L, Tanzer MC, Hermann-Kleiter N*, et al.* Loss of PIDD limits NF-kappaB activation and cytokine production but not cell survival or transformation after DNA damage. *Cell death and differentiation* 2013, **20**(4)**:** 546-557.
